# Land use change and soil salinization in the Sundarbans: a machine-learning based analysis of long-term transformation and future projections

**DOI:** 10.1007/s10661-025-14829-2

**Published:** 2025-11-28

**Authors:** Uttam Kumar Mandal, Amit Ghosh, Fazlul Karim, Sonali Mallick, Dibyendu Bikas Nayak, Rinchen Nopu Bhutia, Ajay Kumar Bhardwaj, Tashi D. Lama, Dhiman Burman, Priyanka Choudhury, Kshirendra Kumar Mahanta, Shishir Raut, Subhasis Mandal, Mohammed Mainuddin

**Affiliations:** 1 ICAR-Central Soil Salinity Research Institute, Regional Research Station, Canning Town, 743 329 India; 2 CSIRO Environment, Canberra, ACT 2601 Australia; 3 ICAR-Central Soil Salinity Research Institute, Karnal, 132 001 India; 4 Dairy Economics Statistics and Management, ICAR-National Dairy Research Institute, Karnal, 132 001 India

**Keywords:** Soil salinity, Vulnerability, Climate change, Erosion-accretion, CA-ANN, MOLUSCE

## Abstract

Quantitative data on coastal land use changes are essential for effective resource management and sustainable development. In this study, we examined land use and land cover (LULC) changes, along with erosion and accretion, in the climate-sensitive Sundarbans of India using satellite imagery from 1973 to 2022. Over this period, approximately 478 km^2^ of land was lost to erosion, while 408 km^2^ were gained through accretion. Although single cropping remains the predominant land use system, the areas dedicated to settlements and aquaculture grew 7 times and 2.4 times, respectively. The double-cropped area expanded by 75.8%, while the mangrove area remained unchanged. Among all land use categories, single-cropped land was found to be the most vulnerable to loss. The soil salinity area of the arable land, calculated using the canopy response salinity index (CRSI) from satellite imagery, rose from 2246.2 km^2^ to 2669 km^2^ in the 30 years since 1989. Using a machine learning algorithm (cellular automata-artificial neural network (CA-ANN)), which incorporated both anthropogenic factors and projected temperature and rainfall data as explanatory variables, we estimated that by 2049, the settlement area will increase by 31.6%, aquaculture will expand by 30%, and vegetation cover will decrease by 12.6%, compared to 2019 levels. The LULC change trend and coastline dynamics are expected to further exacerbate land degradation as the model predicts an increase in soil salinity by 5% over the same period. The results help farmers and policymakers to develop effective land management plans to enhance community readiness and resilience against vulnerabilities.

**Supplementary information:**

The online version contains supplementary material available at 10.1007/s10661-025-14829-2.

## Introduction

Coastal areas worldwide have undergone rapid and significant transformations in shoreline and land use in recent decades, driven by anthropogenic pressures and the accelerating impacts of climate change. These transformations have led to widespread land degradation and the loss of vital ecosystem services (Lal, 2022). Various factors contribute to the changing dynamics of land in coastal regions, including coastal compaction and subsidence, sea level rise (SLR), saltwater intrusion, changes in rainfall patterns, frequent tropical cyclones, and fluctuations in freshwater flow into the sea (Day et al., 2008; Lespinas et al., 2010; Paul et al., 2023). These environmental alterations have had profound effects on natural hydrological systems, geomorphology, and ecological conditions. Human activities have exacerbated the imbalance in sediment transport equilibrium, leading to accretion and erosion processes along the coastline.

The Sundarbans, a pristine eco-region, represents one of the world’s most extensively damaged coastal landscapes on earth. It is located at the confluence of the Ganga-Brahmaputra-Meghna (GBM) river systems. It spreads over approximately 1 million hectares, with the Indian Sundarbans covering 40% of its area, while the remaining part belongs to Bangladesh (Ghosh et al., 2015).

Because of its specific geographical positions, the entire Sundarbans faces constant threat from cyclonic storm surges, tidal flooding, and the shifting courses of its distributary channels within its active delta system. The coastal impact in this region is further exacerbated by a combination of biophysical and socio-economic factors, including high population density, low-lying and flat topography, shallow offshore bathymetry, limited public awareness, inadequate emergency preparedness, and the absence of robust protective infrastructure (Mandal et al., 2019). The SLR in West Bengal coast is one of the highest, ranging from 2.06 to 7.48 mm per year (Mandal et al., 2018).

The lands in these areas suffer from severe degradation due to saline water intrusion from storm surges during cyclones, compounded by a near-surface brackish groundwater table influenced by proximity to the sea, which leads to the capillary rise of saline water during the dry season and further increases soil salinity. Coastal agricultural development in the Sundarbans region is severely constrained by a combination of environmental and agronomic challenges. Coastal agricultural development faces challenges from soil salinity during the dry season, heavy soil texture, low fertility, a short winter growing period, and inadequate drainage management leading to extensive water logging in the low-lying agricultural fields. Additionally, subsistence farming practices and extreme climatic conditions significantly exacerbate land degradation and accelerate the deterioration of soil quality.

Although the coastal delta is both critically important and highly vulnerable, there is a notable paucity of comprehensive studies addressing the spatiotemporal dynamics of land use and the combined effects of human and natural disturbances on its ecosystem (Rahman et al., 2011). Coastal erosion poses a significant challenge along the Indian coast, similar to other maritime nations worldwide, yet there is currently no systematic documentation of shoreline changes in coastal India (Ratheesh et al., 2023). It is crucial to have an inventory of land use and land cover (LULC) dynamics and shoreline changes for effectively planning and implementing sustainable development initiatives in coastal regions. Given the impending challenges posed by climate change, including projected SLR, heightened wave activity, and an anticipated increase in tropical cyclones, an expected overview of the LULC and soil salinity is necessary to conserve the ecological integrity of the Sundarbans. Although existing studies on land accounting have primarily focused on historical land changes, limited attention has been given to future scenarios (Huang et al., 2024). These future projections are crucial in formulating strategies to safeguard this region against the adverse impacts of climate change and ensuring its long-term sustainability.

Satellite-based remote sensing provides a crucial source of information for tracking land use changes over time (Islam et al., 2016; Saha et al., 2024). As large parts of the Sundarbans have been designated as a biosphere reserve and are now inaccessible due to government regulations, remote sensing has become an essential tool for monitoring ecosystem changes and provides a valuable means to refine and update existing land use maps (Quader et al., 2017).

Giri et al. (2007) analyzed the mangrove dynamics of the Sundarbans using Landsat imagery spanning from 1973 to 2000, and their findings showed that over the course of their 25-year data, there had been no substantial changes to the forested area. Rahman et al. (2011) used Landsat images from 1973 to 2010 and reported a net erosion of 170 km^2^ in Sundarbans coastline over 37 years. In a similar study, Islam et al. (2016) used Landsat images from 1989 to 2000 and 2010 and reported a substantial increase in shrimp cultivation with a conversion of mono-cropland and a significant growth of settlement areas in the Ganges tidal floodplain. DasGupta et al. (2019) also reported a substantial expansion of aquaculture areas in the Indian Sundarbans. In a separate study, Paul et al. (2023) analyzed erosion and accretion patterns along the tidal rivers from 1920 to 2020. They reported that the banks of the rivers in the west experienced accretion from 6 to 43 km^2^, while the eastern rivers showed a loss of land from 13 to 52 km^2^ over the past century. However, there is a lack of systematic research on erosion, accretion, and land use dynamics in the Indian part of the Sundarbans considering entire creeks and estuaries of the region, as it is pivotal for effective land management and planning strategies in this vulnerable region. Understanding spatiotemporal dynamics of LULC changes and simulating future scenarios offers valuable insights into both current trends and potential development pathways. To forecast these changes, researchers increasingly rely on various land use prediction models. Among them, the CA-ANN model implemented within MOLUSCE (Modules of Land Use Change Evaluation), has demonstrated effectiveness in forecasting future LULC and identifying vulnerable areas (Kamaraj & Rangarajan, 2022; Muhammad et al., 2022).

Over time, the problem of salinity has become a threat in the region due to more frequent storm surges that cause saline seawater intrusion, as well as the overexploitation of groundwater for irrigation to intensify agriculture. Because farming is no longer profitable and the fishery sector is growing steadily, many agricultural lands are being converted into commercial brackish water farms, further aggravating soil salinization in the region. Given the highly dynamic nature of coastal salinity, which is likely to worsen with projected climate change, continuous monitoring of soil salinity in the region is essential for addressing these challenges.

Hence, the objectives of this investigation are to assess the long-term changes in erosion–accretion and the spatial pattern of LULC within the Indian part of the Sundarbans using satellite imagery. We have studied erosion–accretion alongside LULC because of the region’s unique deltaic location and to understand how natural processes and anthropogenic activity interact to shape the coastal ecosystem. We also explored how these changes impact surface soil salinity in this region. For soil salinity assessment, we used canopy response salinity index (square value) as its indicator. Further, we predicted the LULC and salinity scenario of the region using CA-ANN algorithms during 2049. The uniqueness of the study lies in utilizing CA-ANN for LULC and soil salinity forecasting for the highly dynamic, climatically vulnerable Sundarbans region, where historical and projected climate data, along with other biophysical environmental variables, are used as driving factors. The information from this study will be helpful for local planners, administrative departments (such as agriculture and fishery), policymakers, researchers, and other stakeholders involved in the region’s development and assist in identifying the vulnerable areas so that ameliorative measures can be taken in advance before permanent degradation.

## Materials and methods

### Study area: Indian Sundarbans

**Fig. 1:**
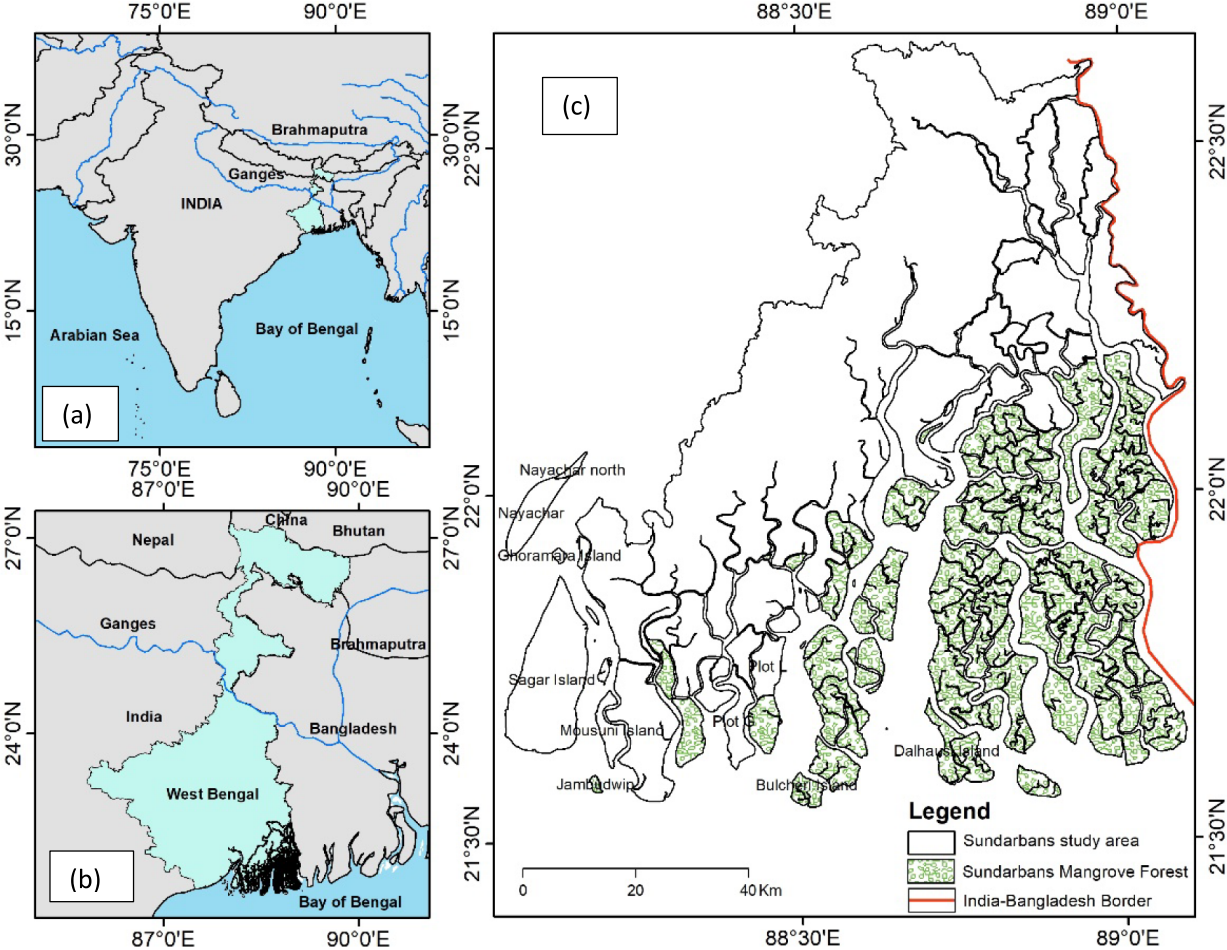
Location map of the study area (Indian Sundarbans): **a** Indian subcontinent and river Ganges and Brahmaputra; **b** West Bengal; **c** Indian Sundarbans

Our study site included the Indian part of the Sundarbans, a tropical region spanning latitudes 21°31′46″N to 22°39′31″N and longitudes 88°02′14″E to 89°06′58″E. It covers 19 administrative blocks—13 in South 24 Parganas district and 6 in North 24 Parganas district of West Bengal, India (Fig. 1). Since 1970, the Sundarbans mangrove has been safeguarded under various legal provisions mainly to protect the endangered royal Bengal tiger and to conserve the mangrove forest. The delta has evolved by natural deposition of upstream sediments, the millennia, from the GBM river systems accompanied by intertidal segregation. Many tidal saline water rivers crisscross the region with the only freshwater flow being within river Hooghly on the west, affecting the salinity levels in the soil and water. While embankments have been constructed along the majority of the areas of Sundarbans to prevent inundation, the mangrove coastline of the region remains un-embanked to protect its unique ecosystem. The Sundarbans has a moist sub-humid climate, characterized by a moderate amount of rainfall throughout the year. The region receives an average annual rainfall of about 1800 mm with monsoon months (June–September) accounting for 75% of it. The soils are poorly drained, heavy-textured, deep, and free from gravel and hardpan, and salinity appears as the dry season progresses.

#### Landsat images data

Since the study aimed to delineate the erosion and accretion, LULC changes, and soil salinity, it was crucial to obtain cloud-free images over the areas of interest. Images from 1973, 1989, 2002, 2011, 2015, 2019, and 2022 from January to April were suitable as they met the criteria of being free from cloud cover. These images were obtained from the archive of the US Geological Survey (USGS) Earth Explorer website (https://earthexplorer.usgs.gov) at no cost. The earliest available image from the Landsat was from 1973 for that region. The region experienced four consecutive devastating cyclone hazards: *Fani* and *Bulbul* in 2019, *Amphan* in 2020, and *Yaas* in 2021. A close observation of the imagery between 2019 and 2022 was needed to observe the impact of recent cyclones in the region. Details of the images regarding their sensors, satellite platforms, and other related information are presented in Table 1. Two adjacent Landsat images were used for studying the entire Sundarbans region of India. Sundarbans fall within Universal Transverse Mercator (UTM) zones 45 N.

**Table 1:** Description of Landsat satellite images used in this study

Platform	Sensor	Year	Band used	Path/row	Resolution (m)
Landsat-1	MSS	1973	4, 5, 6, 7	148/44&45	60
Landsat-5	TM	1989	1, 2, 3, 4, 5, 6, 7	138/44&45	30
Landsat-7	ETM+	2002	1, 2, 3, 4, 5, 6, 7	138/44&45	30
Landsat-5	TM	2011	1, 2, 3, 4, 5, 7	138/44&45	30
Landsat-8	OLI	2015	1, 2, 3, 4, 5, 6, 7	138/44&45	30
Landsat-8	OLI and TIRS	2019	1, 2, 3, 4, 5, 6, 7, 10, 11	138/44&45	30
Landsat-9	OLI	2022	1, 2, 3, 4, 5, 6, 7	138/44&45	30

Landsat images obtained from USGS were clipped to the district boundary map to extract the study area. To maintain consistency, USGS images were resampled to a uniform spatial resolution of 30 m. Radiometric calibration of all the images was carried out to convert digital number (DN) to at-satellite spectral reflectance as it was essential for multi-temporal studies (Chander et al., 2009).

### Detecting coastline dynamics

Temporal changes in erosion, accretion, and the shoreline were performed through overlay analysis of the Landsat images using ArcGIS 10.5 software. Boundaries were demarcated through on-screen visual interpretation of FCC (false color composite) imagery and digitization. The FCC imagery differentiates land from water in each image. In these false color images, bare soils appeared in shades of brown and vegetated areas in red, while mudflats and sandy beaches were shown in varying shades of white. Since water absorbs infrared radiation, it typically appears in shades of blue or black. However, some areas of muddy water showed a brownish hue. The morphology of the bays and river mouths helped confirm that these areas were indeed water, not soil. To further verify the land–water boundary, the normalized difference vegetation index (NDVI) was created by using the red (R) and near-infrared (IR) bands (Rouse et al., 1973). NDVI values are generally positive for dry land and negative for water bodies (Rahman et al., 2011). Based on these NDVI values and the false color composites, land and water areas were classified to demarcate the coastline.

The study analysed erosion and accretion for several periods: 1973–1989, 1989–2002, 2002–2011, 2011–2015, 2015–2019, 2019–2022, and the entire period from 1973 to 2022. While determining erosion, the “baseline” image was taken from the later year within each interval; in contrast, for accretion, it was taken from an earlier year. The direction of land loss or gain was assessed by measuring changes that occurred perpendicular to a defined section of the coastline in the baseline image. The length of these trajectories, multiplied by the area occupied by each pixel, provided an estimate of the areas of erosion or accretion. The erosion and accretion areas were determined with respect to each of the baseline images across all time intervals. The annual erosion and accretion rates were calculated by dividing the net erosion and accretion areas by the number of years for each interval of the study period.

The coastline was considered stable when the land–water boundary in the two different time frames overlapped. At the same time, coastal regions with a landward shift were classified as eroding coasts, while those with a seaward shift were classified as accreting coasts.

### Land use land cover classification

ERDAS software (Nelson & Khorram, 2018) was used for unsupervised classification of images. Based on our expertise and the available auxiliary data from the ground survey, including Google Earth, we identified a cluster of homogenous pixels representing waterbodies, crop areas, fallow land, forest cover, and other related elements. After being initially categorized into 150 classes, images were re-coded to eight categories of LULC classes: vegetation area, agricultural land as single-cropped and double-cropped areas, waterbodies, mangrove areas, creeks, aquaculture, and settlements. In this region, due to dry season salinity, inadequate drainage conditions, and lack of irrigation facilities, only a single crop can be grown. The double-cropped area represents land, where in addition to *kharif* (rainy season) paddy, *boro* (dry season) paddy, vegetables, and pulses are grown during the dry season with irrigation facilities. The waterbodies consist of freshwater ponds and large canals excavated to store rainwater for irrigation and pisciculture. In contrast, areas under aquaculture are mostly brackish water aquaculture, locally known as “bheries,” where farmers allow saline river water to ingress for fish farming. The temporal LULC information was acquired via interviews with the local people, supplemented by ground verification, historical land use maps, and databases including Google Earth information.

Following the LULC classification, accuracy assessment, and kappa coefficient estimation were carried out. For each image, an error matrix was developed to calculate user accuracy, producer accuracy, and the kappa coefficient of agreement.

### Land use Land cover change analysis

Change analysis of LULC was carried out for monitoring the land dynamics of different study periods. The percentage area of each LULC class in classified images was calculated to know the trends of changes. For the LULC change detection assessment, the raster LULC was converted to polygon and run the intersect tool to detect class-wise changes among the LULC images. The change matrix has been generated from attributes of the merged LULC layer using a pivot table in Excel software. An area change matrix was generated, with rows and columns representing LULC categories for the start and end years of each interval. The result presented the gains and losses in area change by the LULC classes estimated from the transition matrix for different time intervals (Islam et al., 2016).

### Delineating salinity area

The canopy response salinity index (CRSI), as mentioned below, was used for this study to identify the salinity area (Scudiero et al., 2015).

1 $$CRSI=\sqrt{\frac{\left(NIR \times R\right)-\left(G \times B\right)}{\left(NIR \times R\right)+\left(G \times B\right)}}$$

B, G, R, and NIR represent blue, green, red, and near-infrared corresponding to band 1, 2, 3, 4 of TM and ETM+ sensors and band 2, 3, 4, and 5 of OLI sensor. In calculating the salinity index, the square of CRSI (referred to as CRSISQR) was used to eliminate negative CRSI values. A total of 192 georeferenced soil samples were collected from the top 20 cm depth, and the electrical conductivity of saturation paste extract (ECe) of each soil sample was recorded. A relationship was established between CRSISQR and the ECe of observed soil samples, which showed that the ECe is inversely proportional to CRSISQR (Mandal et al., 2023). Based on FAO (2020) classification, soils were classified as non-saline (ECe < 2 dS m⁻^1^), slightly saline (ECe between 2 and 4 dS m⁻^1^), and saline (ECe > 4 dS m⁻^1^). These categories corresponded to CRSISQR values greater than 0.16 for non-saline, between 0.12 and 0.16 for slightly saline, and less than 0.12 for saline soils (Mandal et al., 2023). The salinity values of observation points were grouped into non-saline (ECe < 2dS m^−1^), slightly saline (ECe 2–4 dS m^−1^), and saline (ECe > 4 dS m^−1^), and the corresponding points from the CRSISQR map for 2019 were utilized for creating the confusion matrix to verify the classification accuracy.

### Secondary information of the study area

The present study used a wide range of secondary data (Table 2). Population data was obtained from censusindia.gov.in. Topography data, in the form of a 30 m resolution digital elevation model (DEM), was obtained from the WorldClim database (www.worldclim.org). Proximity to creeks or rivers is the driving force behind the landscape design and soil salinity of coastal regions and is derived by specifying a buffer distance value using ArcGIS buffering tools. Similarly, we also delineated a spatial variable map of distance from the road or proximity to the road by downloading the road network shapefile of our study region from the OpenStreetMap source.

**Table 2:** Source of dataset maps of Indian Sundarbans

Data	Source	Description
DEM	https://worldclim.org	The digital elevation model (DEM) obtained from WorldClim with a spatial resolution of 30 m and was derived from SRTM elevation data
Population density (1991, 2001, 2011)	censusindia.gov.in	Block-wise population data for 1991, 2001 and 2011 was downloaded from Indian Census
Distance from creeks or proximity to creeks	Creeks data digitized manually through visual interpretation of Landsat imagery and verified with google earth database	Derived by specifying a buffer distance value through buffering in ArcGIS using creeks data of the study region
Distance from road or proximity to road	OpenStreetMap	Road network data was downloaded and derived by specifying buffer distance value through buffering in ArcGIS
LST (land surface temperature)	Landsat thermal bands	The land surface temperature (LST) distribution was estimated using Landsat thermal bands; the sixth band of Landsat’s 5 TM and 10 and 11 bands of Landsat 8 TIRS (thermal infrared sensor) sensor
Historical climate data	https://www.ecmwf.int/en/forecasts/datasets/reanalysis-datasets/era5	Satellite based historical gridded data was downloaded for three climatic parameters; average of yearly maximum and minimum value of daily maximum and minimum temperature, and mean annual rainfall for the year 1985–2020
Future climate data	https://esgf.nci.org.au/projects/esgf-nci/	The future climate data for three climate parameters as mentioned in historical climate data for the period 2041–2060 based on IPCC sixth assessment report for scenario SSP585 (Shared Socio-Economic Pathways)

Historical and future climate data for Tmax_mean (average of the yearly maximum daily temperatures), Tmin_mean (average of the yearly minimum daily temperatures), and mean annual rainfall were utilized as explanatory variables in this study. Historical climate data (1985–2020), derived from satellite-based gridded datasets, were obtained from the ERA5 reanalysis dataset through the ECMWF (European Centre for Medium-Range Weather Forecasts) website (https://www.ecmwf.int/en/forecasts/datasets/reanalysis-datasets/era5). We have used future climate data for the period 2041–2060 based on the IPCC sixth assessment report for the SSP585 scenario (Shared Socio-Economic Pathways). These data were sourced from the Earth System Grid Federation portal hosted by the National Computational Infrastructure of Australia (https://esgf.nci.org.au/projects/esgf-nci/) (Karim et. al., 2024; Mandal et al., 2025). We have used the highest climate change scenario (SSP585) to record the extent of change in LULC and salinity prediction.

### Estimation of land surface temperature (LST)

The region’s LST distribution as one of the explanatory variables was estimated using Plank’s law applied to the thermal bands of Landsat imagery. Specifically, band 6 of Landsat 5 TM and bands 10 and 11 of Landsat 8 TIRS (thermal infrared sensor) sensors were used, following the methodology outlined by Kafy et al. (2023) and Kumar et al. (2024).

The following equation was applied to convert radiance into brightness temperature (*T*_B_) in Kelvin:

2 $$T_B=\frac{K2}{\text{ln}(\frac{K1}{L\lambda }+1)}$$

where *L*_λ_ = spectral radiance of the Landsat thermal bands (measured in Wm^−2^sr^−1^ mm^−1^).

*K*_2_ and *K*_1_ are calibration constants; *L*_λ_ was calculated using Eqs. 3 and 4 using values for Landsat TM, *L*_max_, and *L*_min_ recorded in the satellite metadata file.

3 $$L_\lambda\;(LANDSAT\;5\;TM)\:=\:L_{min}\;+\frac{L\text{max}-L\text{min}}{255}\times DN$$

where *DN* is the digital numbers from the thermal bands.

4 $$L_\lambda\;(LANDSAT\;8\;OLI)\;=\;ML\;\times\;DN\;+\;AL$$

where ML is the band specific multiplicative rescaling factor; AL is the band specific additive rescaling factor; and DN is the digital numbers from the thermal bands (Band 10 and 11 for Landsat 8 TIRS).

To estimate surface emissivity (ε), the NDVI thresholding method is applied as described by Sobrino et al. (2001). Fractional vegetation (Fv) for each pixel is derived from the NDVI values using the formula given by Carlson and Ripley (1997):

5 $$Fv=\left(\frac{\text{NDVI}-\text{NDVImin}}{\text{NDVImax }-\text{ NDVImin}}\right)^2$$

where NDVI_min_ is the minimum NDVI value of pixels and NDVI_max_ is the maximum NDVI value of pixels with healthy vegetation cover. The emissivity (*ε*) is calculated from Fv using Eq. 6.

6 $$\varepsilon\;=\;0.004\times Fv+0.986$$

Finally, the LST was derived using Eq. 7.

7 $$LST=\frac{TB}{1+\left(\lambda\sigma\frac{TB}{hc}\right)\text{ln}\;\varepsilon}$$

where *λ* is the effective wavelength (10.9 mm for band 10 in Landsat 8 and 9 and 11.45 mm for band 6 in Landsat 5 data); *σ* is the Boltzmann constant (1.38 × 10^–23^ JK^−1^); *h* is the Plank’s constant (6.626 × 10^–34^ Js); and *c* is the velocity of light in a vacuum (2.998 × 10^8^ ms^−1^). 

### Prediction analysis and validation

A crucial aspect of this study is the prediction of future LULC and soil salinity distribution. Soil salinity was estimated from the CSRISQR index map. This study used CA-ANN (cellular automata-artificial neural network) algorithms to predict the region’s future LULC and soil salinity. The ANN is a machine-learning approach characterized by multiple layers of neurons or nodes, similar in function to a biological nervous system capable of analyzing large datasets to identify patterns (Khalid et al., 2024) and make predictions, whereas CA algorithms divide complex systems into smaller, interconnected cells (Kafy et al., 2021, 2023). For forecasting the spatiotemporal distribution of LULC and soil salinity, the study used the MOLUSCE plugin within QGIS software 2.18.15, which applies CA-ANN algorithms. The plugin comprises several components, including data input, change trend analysis, modeling techniques, prediction, and validation modules. MOLUSCE enables users to simulate categorical changes in land use and land cover in a simplified and unsupervised manner, facilitating spatial prediction and scenario analysis (Kafy et al., 2023). ANN has several advantages in handling complex non-linear data of various bio-physical and environmental variables compared to other traditional approaches (Sun et al., 2024).

The modelled results were validated using an existing LULC database, followed by an accuracy assessment. After a satisfactory match between the simulated LULC and observed data, the model was used to simulate future LULC scenarios using the calibrated settings. LULC layers of two distinct study periods were used as dependent parameters for each simulation analysis. Both natural and anthropogenic factors have a substantial influence on LULC change. The selection of these variables was based on their contextual relevance to the study area, data availability, and insights from the literature review. The spatial driving factors for land surface dynamics, such as DEM, population density, NDVI, and CRSI (in our study, it is CRSISQR) for the two different study periods, distance from creeks, distance from the roads, and climate data (Tmax_mean, Tmin_mean, and annual rainfall) were treated as independent variables. Kamaraj and Rangarajan (2022) used DEM, slope, aspect, distance from the road, and distance from built-up areas as spatial environmental variables for predicting future LULC. Similarly, Kafy et al. (2023) monitored spatio-temporal dynamics of agricultural drought in the Barind Tract region of Bangladesh using various spectral vegetation indices, along with rainfall and soil moisture data. Intersect tools in ArcGIS were used to create a common boundary of all the explanatory maps for different years, and all maps were extracted in the same raster format to run MOLUSCE.

A potential transition matrix of LULC change was generated by analyzing the correlations of input layers and area change between the study periods. The CA-ANN model optimizes accuracy based on two sequential LULC layers with supporting raster layers. The correlation of geographic variables between raster images is evaluated using the Spearman correlation matrix. The classified images of 1989 and 2002 were used to predict the LULC of 2015; then, the predicted 2015 images were validated with the classified 2015 images. A range of explanatory variables like DEM, NDVI, and CRSISQR for 1989 and 2002, population density data from 1991 and 2001 and distance from creeks, proximity to roads, and historical climate variables such as Tmax_mean, Tmin_mean, and annual rainfall were used as independent variables. These variables served as independent inputs for the LULC simulation. The projected 2015 salinity map was created in MOLUSCE from the salinity map of 1989 and 2002, and the explanatory independent variables were DEM, NDVI, and LST for 1989 and 2002, distance from creeks, and historical climate data (Tmax_mean, Tmin_mean, and annual rainfall). The projected 2015 salinity map was validated with the actual salinity map of 2015 generated from the CRSISQR of 2015.

**Fig. 2:**
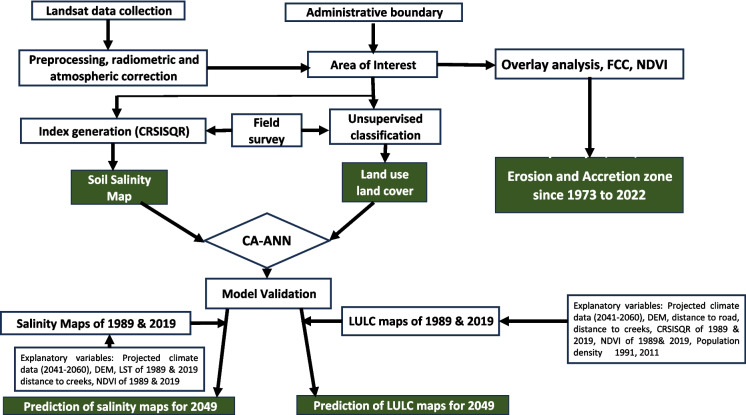
Flow chart of the methodology

For the projected LULC of 2049, the LULC maps of 1989 and 2019 were used as dependent variables and DEM, NDVI, and CRSISQR for the years 1989 and 2019, the population density of 1991 and 2011; distance from creeks, distance from the roads, and future climate data (Tmax_mean, Tmin_mean, and annual rainfall) were used as independent variables. Similarly, for the projected salinity map of 2049, salinity maps of 1989 and 2019 generated from CSRISQR were used as dependent variables and DEM, LST of 1989 and 2019, distance to creeks, and NDVI of 1989 and 2019, and future climate data were used as independent variables. The overall methodological framework is illustrated in Fig. 2.

The CA-ANN model, used for projecting LULC and soil salinity, is subject to uncertainties from model structure, component interactions, and input data quality (Naeem et al., 2025). To mitigate these uncertainties, we used bias-corrected climate datasets to reduce input errors (Kamruzzaman et al., 2021). To project the LULC and soil salinity in the ANN learning process, a learning rate of 0.001, a neighborhood value of 3 × 3 pixels, 12 hidden layers, 0.001 momentum, and 100 iterations were chosen after several trials and errors, till the minimum validation overall error was found to < 0.05. Further, to evaluate the potential explanatory power of the variables and to measure the association between LULC classes and salinity with the driving environmental factors/variables, Cramer’s V was computed for all the explanatory variables. Model accuracy was evaluated using the Kappa coefficient and the area under the receiver operating characteristic (ROC) curve (AUC), both of which were employed for model validation. To validate the model results, the projected LULC of 2015 was compared to the classified LULC for 2015. During the validation stage in MOLUSCE, a kappa coefficient for the projected LULC of 2015 was produced in comparison to the actual LULC layer of 2015. Similar criteria were chosen for salinity map projection and validation. The projected (or simulated) maps were used to calculate the ROC statistics, with the AUC values indicating the accuracy of the model. Higher AUC values reflect greater model accuracy and reliability, whereas lower values suggest reduced predictive performance.

## Results

### Erosion and accretion of the region

**Fig. 3:**
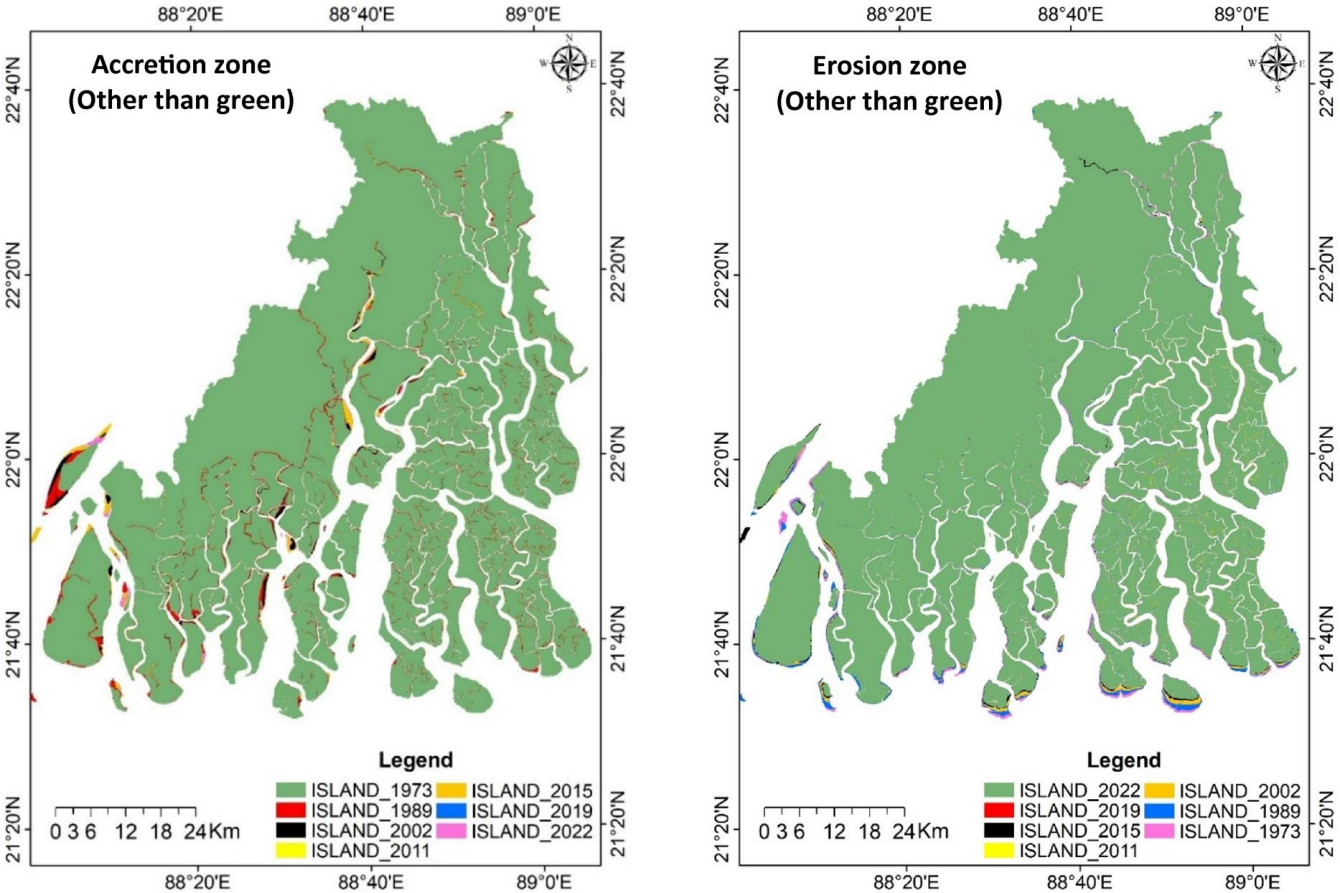
Erosion and accretion zone (other than green) of Indian Sundarbans between 1973 and 1989, 1989 and 2002, 2002 and 2011, 2011 and 2015, 2015 and 2019 and 2019 and 2022 based on Landsat imagery

The erosion and accretion rates varied throughout the selected time intervals as shown in Table 3, and its spatial distribution of the study area is depicted in Fig. 3. During 1973 to 2015, the erosion rate was significantly high, over 10 km^2^ yr^−1^. However, it declined to 2.7 km^2^ yr^−1^ during 2015–2019 but again increased to 6.8 km^2^ yr^−1^ during 2019–2022. Accretion followed a similar trend, with the highest rate observed during the initial period of study (1973–1989). It remained relatively consistent at around 6–8.5 km^2^ yr^−1^ from 1989 to 2015, declined to 3.8 km^2^ yr^−1^ during 2015–2019, and increased to 6.6 km^2^ yr^−1^ during 2019–2022. Over the six distinct periods, a total of 478 km^2^ of land was lost to erosion, where 408 km^2^ was gained through accretion. Among the six study periods, four experienced a net loss of land due to erosion, ranging from 0.6 to 53 km^2^. During 1973–1989 and 2015–2019, there was a net gain in land, amounting to 4 to5 km^2^. Overall, land dynamics remained relatively stable during 2015–2019.

**Table 3:** Accretion and erosion in the Indian Sundarbans in the period of 1973–2022

Period	Total accretion (km^2^)	Total erosion (km^2^)	Accretion (km^2^ yr^−1^)	Erosion (km^2^ yr^−1^)	Difference (km^2^)
1973–1989	174.09	168.87	10.88	10.55	5.22
1989–2002	88.10	141.69	6.78	10.90	−53.59
2002–2011	77.04	94.66	8.56	10.52	−17.62
2011–2015	33.82	41.86	8.45	10.46	−8.04
2015–2019	15.34	10.95	3.83	2.74	4.39
2019–2022	19.80	20.41	6.60	6.80	−0.61
Total	408.18	478.44	8.33	9.76	−70.25
1973–2022	269.12	305.58	5.49	6.24	−36.46

**Fig. 4:**
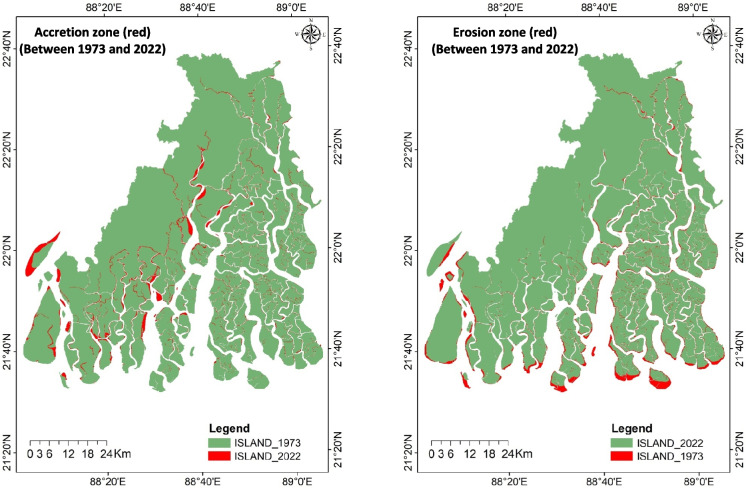
Erosion and accretion zone (red) of Indian Sundarbans between 1973 and 2022 based on two Landsat imagery pair

Comparing land dynamics using the 1973 and 2022 image pair revealed a significant discrepancy in total erosion and accretion over the 49 years compared to the values from the six intervals (Fig. 4 and Table 3). The total erosion and accretion calculated across the six intervals were about 1.5 times higher than those observed from the 1973 and 2022 images. This indicates that both erosion and accretion were highly dynamic throughout the study period.

**Fig. 5:**
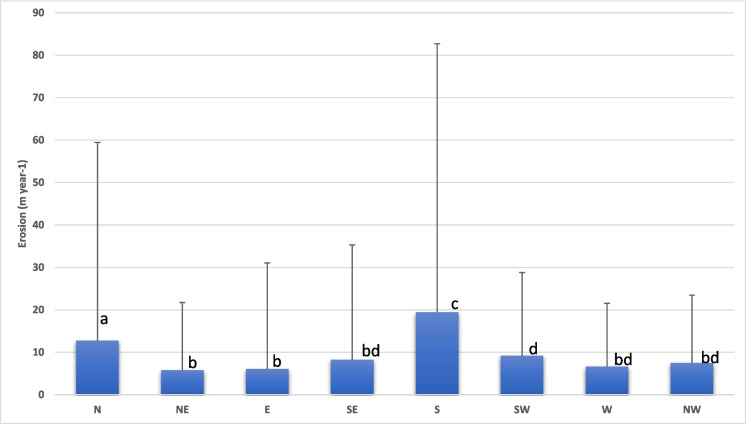
Erosion rate (m year^−1^) of the Indian part of the Sundarbans coastline estimated using 1973 and 2022 Landsat images, the thick blue bar represents the average value, and the thin line represents the maximum value (thick bar values followed by the same letters are not significantly different at *p* < 0.05); N, NE, E, SE, S, SW, W, and NW are the directions of erosion of the Sundarbans islands towards North, North East, East, South East, South, South-West, West, and North-West, respectively

**Fig. 6:**
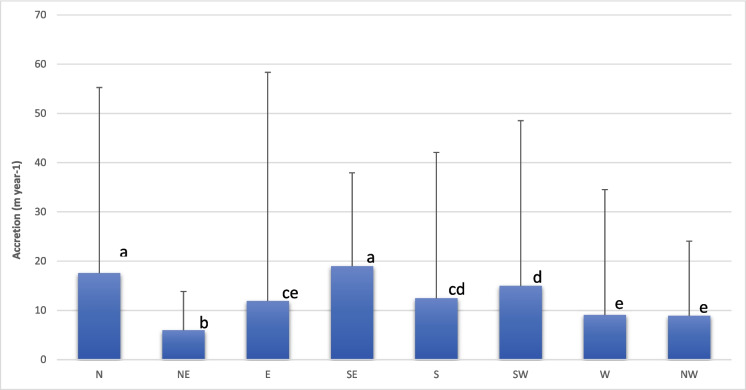
Accretion rate (m year^−1^) of the Indian part of the Sundarbans coastline estimated using 1973 and 2022 Landsat images; the thick blue bar represents the average value, and the thin line represents the maximum value (thick bar values followed by the same letters are not significantly different at *p* < 0.05); N, NE, E, SE, S, SW, W, and NW are the directions of accretion of Sundarbans islands towards North, North East, East, South East, South, South-West, West, and North-West, respectively

The study identified an average linear coastal erosion rate of 9 m yr^−1^ across all azimuthal directions over the entire study period, as illustrated in Fig. 5. Notably, the mean erosion rates were 19 m yr^−1^ in the south, 10.3 m yr^−1^ in the north, 6.5 m yr^−1^ in the west, and 6.1 m yr^−1^ in the east directions. Maximum erosion was observed at 63 m yr^−1^ on the southern edge of island L Plot of Sundarbans. On the other hand, average accretion was also similar; it was 17.5 m, 12.4 m, 11.9 m, and 9 m, respectively, in the north, south, east, and west directions (Fig. 6). The spatial change analysis was conducted along the shoreline, encompassing the mouths of rivers and creeks as well as their inner regions. The Indian Sundarbans extends 12,601 km of shoreline, including river mouths, streams, creeks, and island boundaries and adjacent interior areas. The analysis revealed that between 1973 and 2022, about 5643 km (44.8%) of the shoreline experienced erosion, 5394 km (42.8%) showed accretion, and only 1564 km (12.4%) remained stable, underscoring the highly dynamic nature of the Sundarbans.

### Trend of land surface area of Sundarbans islands

We studied the land surface dynamics of the islands in the Indian Sundarbans during 1973–2022. Out of 47 islands, the land surface area of 11 islands is highly dynamic in the Indian Sundarbans (Table 4). Bedford Island had already disappeared after 1989 from its original location. More than half the area of the Jambudwip and Ghoramara islands vanished within 49 years of our study. The populous Sagar Island showed 1% growth in the area from 1973 to 2022, whereas 2.3% of the area disappeared from 1989 to 2022. Around 7.9 km^2^ area of the island experienced sedimentation between 1973 and 1989. Though most of the islands’ areas are eroding, Nayachar Island in the river Hooghly has grown more than 52% between 1973 and 2022. Some of the islands are densely populated, and displacement is a great challenge in the Sundarbans region.

**Table 4:** Temporal variation in land surface area (km^2^) of selected islands in the Indian Sundarbans

Island names	1973	1989	2002	2011	2015	2019	2022	% Change (2022–1973)	Population (2011)*
Bedford Island	4.6	1.3							
Bulcheri Island	30.1	27.5	23.7	21.8	20.7	20.7	19.0	−37.0	Reserved forest
Dalhausi Island	77.1	71.7	63.3	59.8	57.3	57.3	57.3	−25.7	Reserved forest
Ghoramara Island	7.9	6.1	4.6	4.3	4.2	3.8	3.5	−56.4	5193
Jambudwip	6.8	7.7	4.2	4.2	3.9	3.9	3.3	−51.9	No habitation
Mousuni island	31.8	30.7	27.7	27.8	27.1	26.9	26.8	−15.8	22,073
Nayachar	31.9	41.0	47.6	44.8	48.4	48.9	48.6	52.2	2500
Nayachar north			0.5	3.2	3.0	4.9	5.5		Not available
Plot G	48.2	44.9	40.9	40.3	40.3	41.1	41.4	−14.1	No habitation
Sagar Island	230.3	238.2	230.3	234.4	235.2	233.6	232.7	1.0	206,844
L Plot	36.4	35.3	33.6	33.4	32.8	32.9	33.0	−9.1	No habitation

*Census of India, 2011

### Land use land cover study

#### Classification accuracy of LULC

**Fig. 7:**
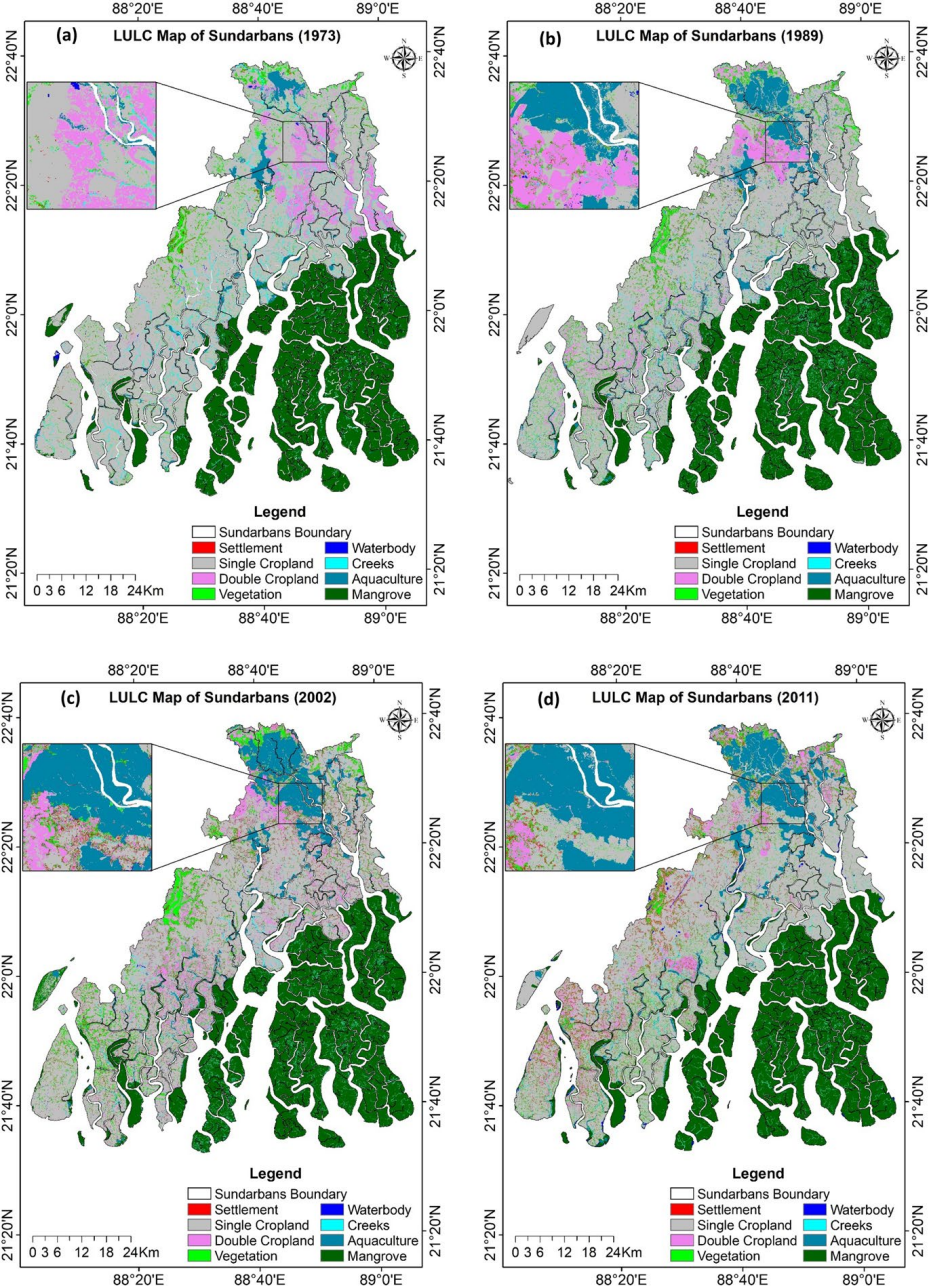

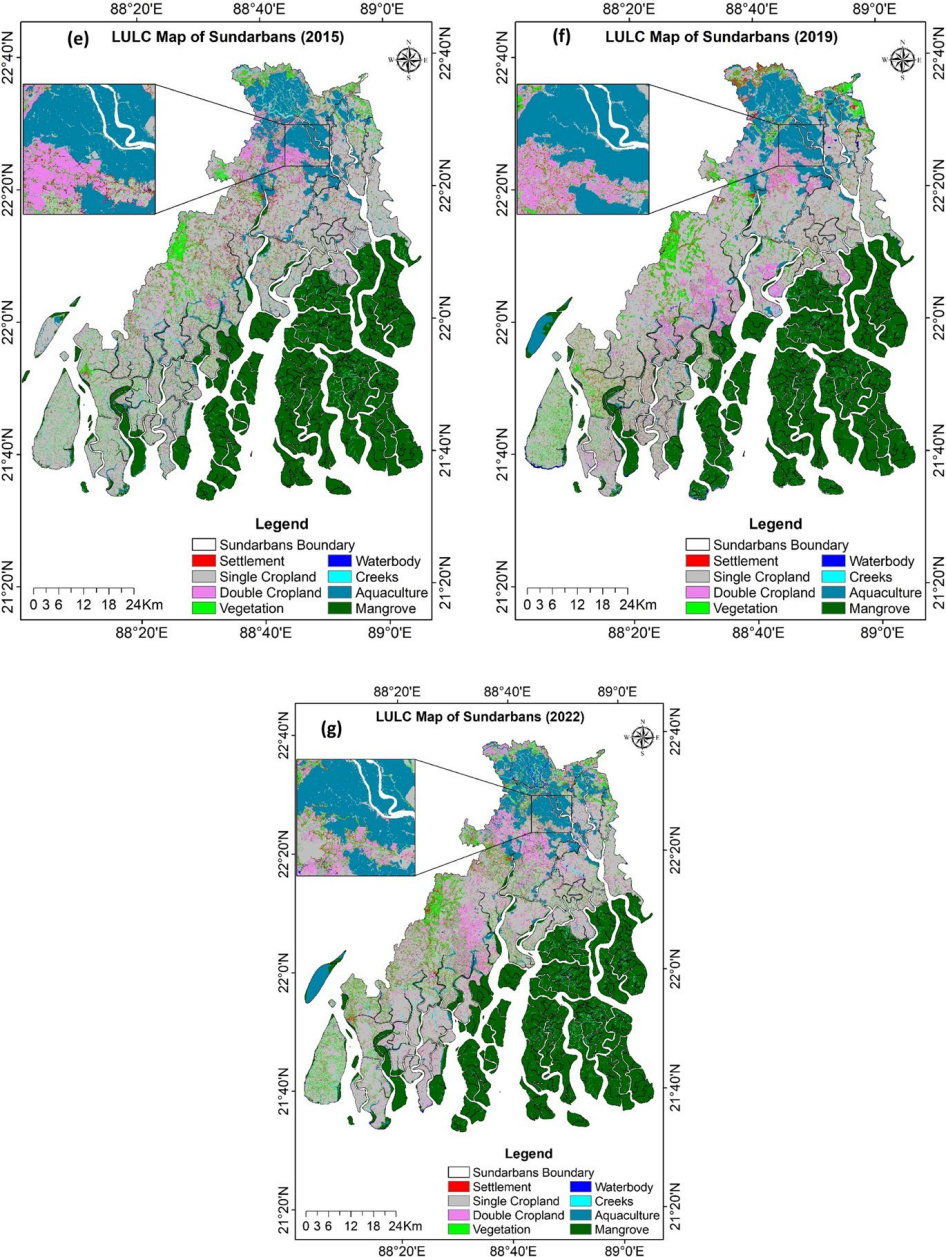
Land use and land cover (LULC) map of Indian Sundarbans for the year **a** 1973, **b** 1989, **c** 2002, **d** 2011, **e** 2015, **f** 2019, **g** 2022

The LULC maps, derived from Landsat imagery for the years 1973, 1989, 2002, 2011, 2015, 2019, and 2022, are presented in Fig. 7a to g. By loading the predefined training or test area mask, the LULC classification output was verified, and its accuracy level was calculated (Table 5). The overall classification accuracies were 0.86, 0.89, 0.90, 0.89, 0.85, 0.90, and 0.91 for the years 1973, 1989, 2002, 2011, 2015, 2019, and 2022, respectively. The kappa coefficients for these classifications ranged from 0.83 to 0.88. For individual LULC classes, user’s accuracy ranged from 0.67 to 1.00, while producer’s accuracy ranged from 0.65 to 1.00. The classification accuracy levels and corresponding kappa indices of the LULC maps indicated adequate reliability and consistency, confirming their suitability for conducting quantitative analyses of long-term LULC changes.

**Table 5:** Accuracy assessment of land use land cover classes of Indian Sundarbans

Land classes	*U’s_Acc	**P’s_Acc	U’s_Acc	P’s_Acc	U’s_Acc	P’s_Acc	U’s_Acc	P’s_Acc	U’s_Acc	P’s_Acc	U’s_Acc	P’s_Acc	U’s_Acc	P’s_Acc
	1973		1989		2002		2011		2015		2019		2022	
Settlement	0.90	1.00	0.90	0.90	0.88	1.00	0.80	1.00	0.78	0.78	0.71	1.00	0.86	0.86
Double cropland	0.83	1.00	0.90	0.82	0.83	1.00	0.80	0.89	0.90	1.00	0.73	1.00	0.75	1.00
Single cropland	0.92	0.69	0.93	1.00	0.92	0.92	0.92	0.92	0.83	0.77	0.92	0.79	0.89	0.94
Vegetation	0.90	0.69	0.90	0.75	1.00	0.65	0.88	0.70	1.00	0.82	1.00	0.92	1.00	0.75
Aquaculture	0.90	0.90	1.00	0.90	1.00	1.00	0.90	0.90	0.90	1.00	1.00	0.86	1.00	0.85
Waterbody	0.71	1.00	0.78	0.78	0.86	0.86	0.80	0.89	0.70	1.00	0.90	0.90	1.00	1.00
Mangrove	0.80	1.00	0.89	1.00	0.67	1.00	1.00	1.00	0.91	0.83	1.00	0.88	1.00	1.00
Creeks	0.89	0.89	0.78	1.00	1.00	1.00	1.00	0.83	0.78	0.70	0.90	1.00	1.00	1.00
Overall Accuracy		0.86		0.89		0.90		0.89		0.85		0.90		0.91
Overall Kappa		0.84		0.87		0.88		0.87		0.83		0.88		0.88

*U’s_Acc, user’s accuracy; **P’s_Acc, producer’s accuracy

#### Changing trend of LULC

Table 6 shows the area covered by each individual land class derived from the classified LULC maps. Over the study period from 1973 to 2022, the dominant land-use system was single cropland, whereas the waterbody was the least occupied area. Although monocropping (single crop) was the most prominent LULC in 1973, occupying 50.8% of the total land area, its extent declined to 37.5% by 2022, with an average annual loss of approximately 16.4 km^2^. The area under double cropping increased from 4.7 to 8.2% during the same period. At the same time, the mangrove area remained almost unchanged, occupying 33–34% of the total land area throughout the 49-year period. The aquaculture area, mostly under brackish water, also more than doubled from 3.6 to 8.7%. The settlement, along with the vegetation area, also increased from 0.44 to 3.07% and from 3.07 to 7.01%, respectively, from 1973 to 2022. The creeks area decreased from 2.71 to 1.07% due to the accretion of the sediments. In contrast, waterbodies, mostly freshwater ponds and water harvesting structures, increased marginally from 0.21 to 0.61% due to land excavation.

**Table 6:** Area (km^2^ and % of total area) covered under different land use land cover types of Landsat images for 1973, 1989, 2002, 2011, 2015, 2015, 2019, and 2022

LULC Classes	Year 1973		Year 1989		Year 2002		Year 2011		Year 2015		Year 2019		Year 2022		
	Area (km^2^)	% area	Area (km^2^)	% area	Area (km^2^)	% area	Area (km^2^)	% area	Area (km^2^)	% area	Area (km^2^)	% area	Area (km^2^)		% area
Aquaculture	225.01	3.64	384.01	6.10	459.32	7.38	490.91	7.84	514.09	8.23	533.83	8.55	541.08		8.66
Creeks	167.39	2.71	161.11	2.56	132.81	2.13	88.74	1.42	83.18	1.33	63.19	1.01	67.10		1.07
Double cropland	290.46	4.70	303.96	4.83	466.27	7.50	468.05	7.48	448.32	7.18	495.06	7.92	510.59		8.17
Single cropland	3140.28	50.80	2944.37	46.76	2428.79	39.04	2506.76	40.05	2432.60	38.94	2393.69	38.32	2339.13		37.45
Mangrove	2107.47	34.09	2080.03	33.03	2127.83	34.21	2107.15	33.67	2144.67	34.33	2109.62	33.77	2120.53		33.95
Settlement	27.27	0.44	60.57	0.96	150.42	2.42	169.61	2.71	174.70	2.80	184.64	2.96	191.45		3.07
Vegetation	210.24	3.40	329.41	5.23	419.94	6.75	396.09	6.33	408.01	6.53	429.89	6.88	437.90		7.01
Waterbody	13.06	0.21	33.86	0.54	35.21	0.57	31.38	0.50	40.95	0.66	37.22	0.60	38.41		0.61

#### LULC transition analysis

We calculated the transition matrix over the periods 1973–1989, 1989–2002, 2002–2011, 2011–2015, 2015–2019, 2019–2022, and 1973–2022 based on the existing LULC of the respective years to illustrate how each LULC was changed over time. The LULC change matrix from 1973 to 2022 showed that 17.3%, 13.7%, 5%, 82.6%, 96.4%, 2.7%, 14.6%, and 1.1% of the aquaculture, creeks, double cropland, single cropland, mangrove, settlements, vegetation, and waterbody, respectively, were retained in the same land use categories (Supplementary Table 1).

The most stable land cover categories, exhibiting transition probabilities of greater than 0.82, were mangroves followed by mono-cropped areas, whereas the most dynamic systems, with probabilities of 0.01–0.03, were settlement areas and waterbodies. A similar trend was observed during different periods of study, with mangrove and single cropland systems being the most stable; also, single cropland experienced fragmentation and primarily contributed land to convert to aquaculture, double cropland, settlement, and vegetation (Supplementary Tables 2 to 7). The mangroves contributed a small area to creeks due to the wave action of tidal rivers.

**Fig. 8:**
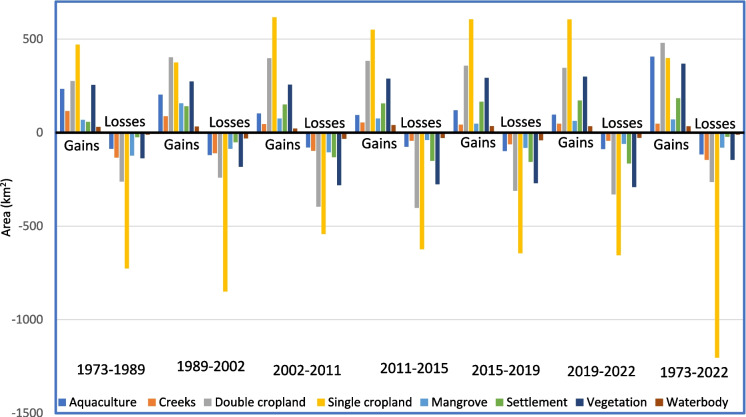
Gains and losses (km^2^) of LULC classes in Indian Sundarbans during 1973–1989, 1989–2002, 2002–2011, 2011–2015, 2015–2019, 2019–2002, and 1973–2022

When we compared the gains and losses of land under different time scales the highest land losses were found for single cropland which was converted to other land uses (Fig. 8). During 1973–2022 around 19.5% of single cropland was lost, in contrast, net gains for it were only 6.4%. In all seven study periods, land losses were maximum for single-cropped land. Again during 1973–89 and 2002–2022, the highest land gain was also achieved in single croplands, whereas during 1989–2002, the double cropland achieved maximum land gain. The study showed that land use dynamics was most prominent in cropland areas. Around 24% of the study area was affected by land use dynamics during 1973–1989, whereas it was 26–27% during the remaining study period. Overall, 32% of the area was influenced by land dynamics between 1973 and 2022.

### Secondary data of study region

**Fig. 9:**
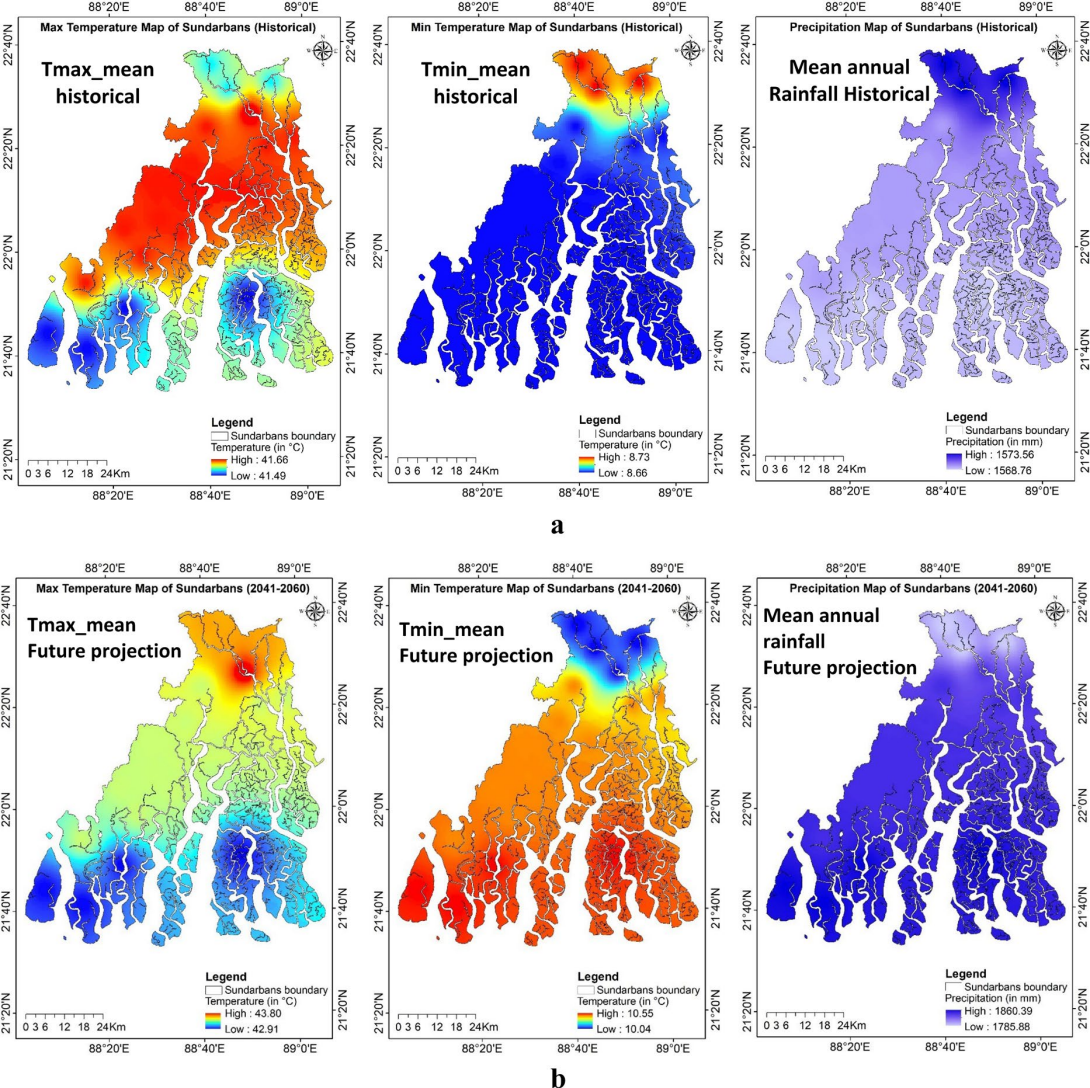

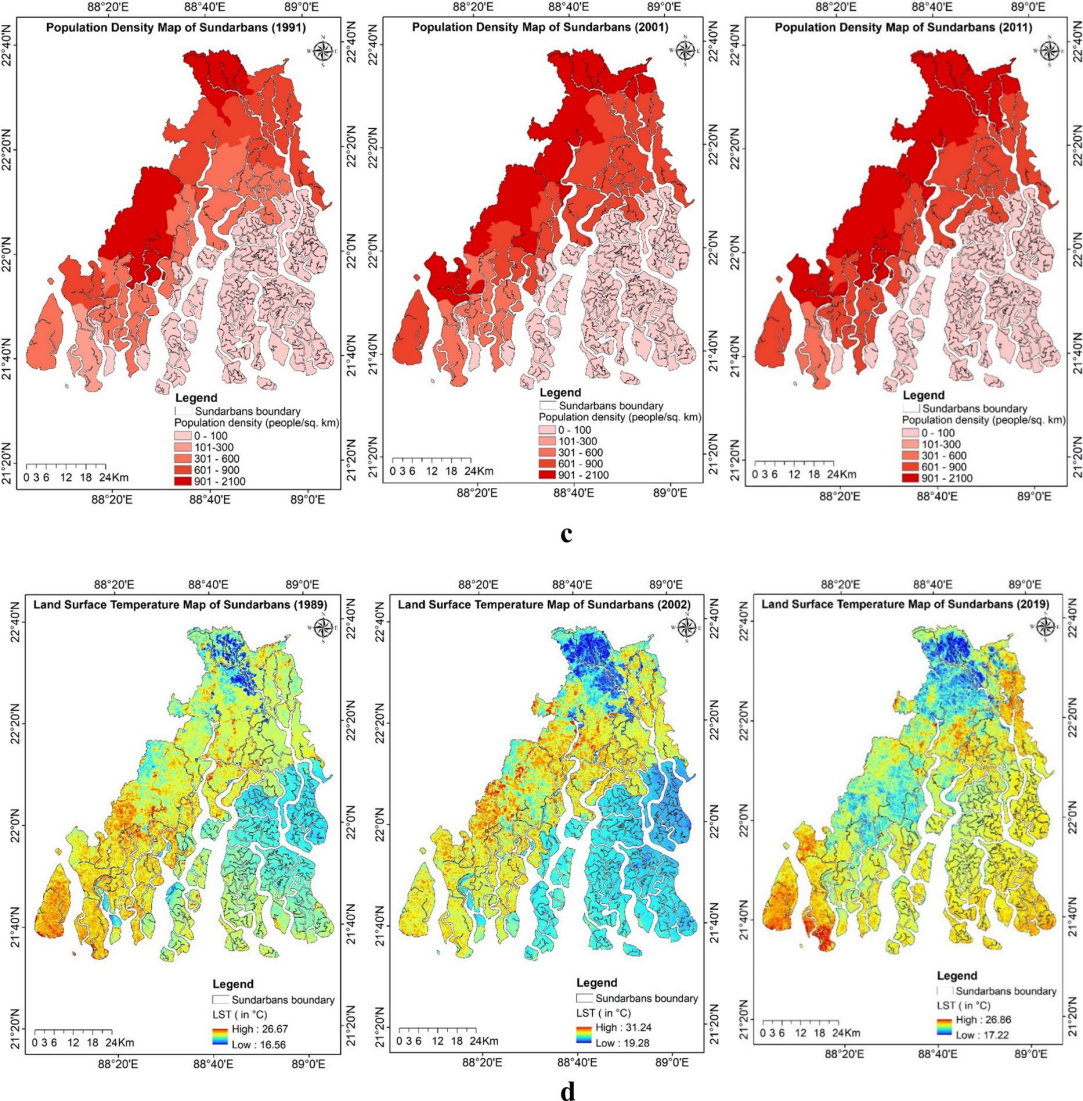

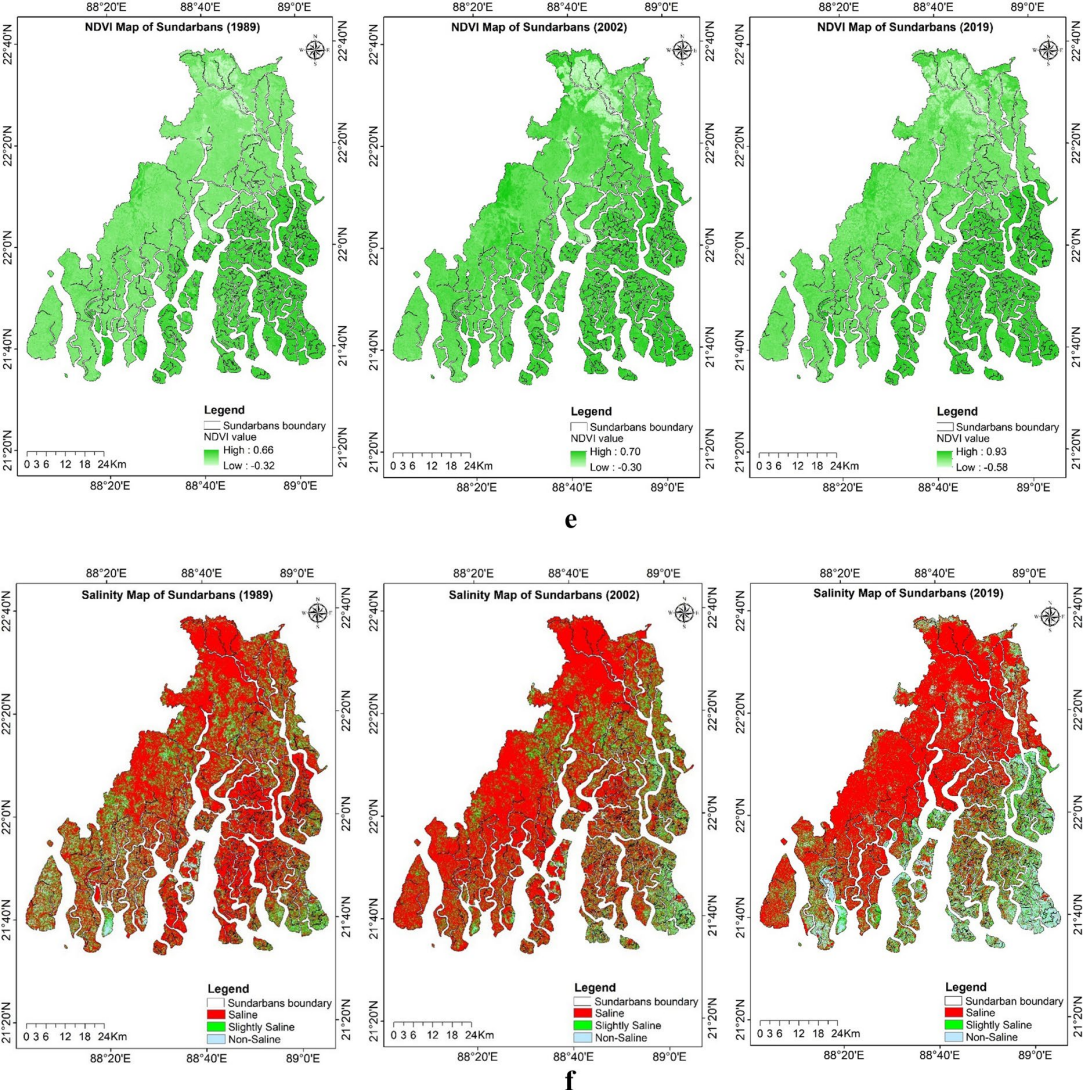

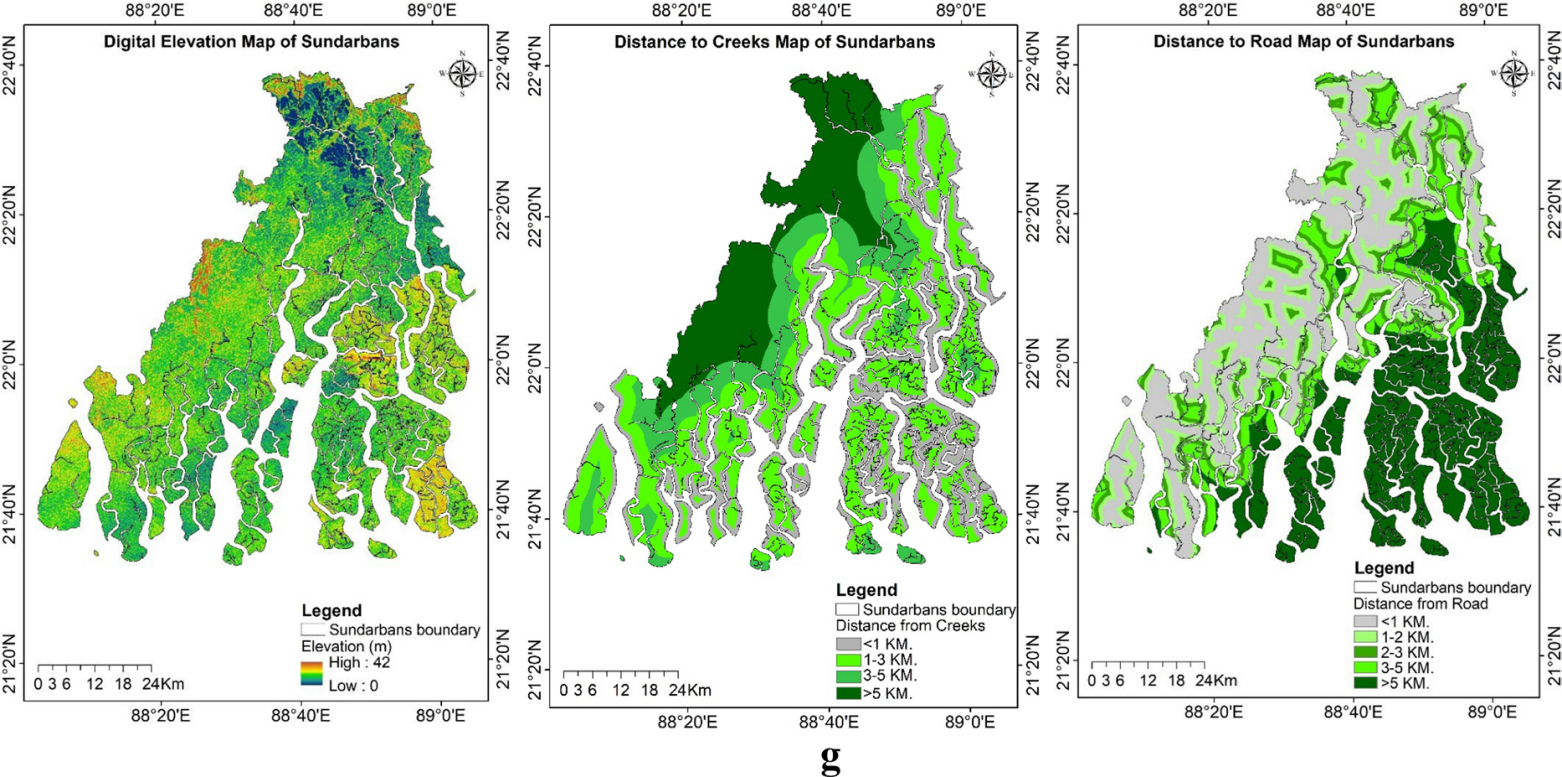
**a** Explanatory variables maps of the study region: historical climate data during 1985–2020, Tmax_mean (average of yearly maximum value of daily maximum temperature), Tmin_mean (average of yearly minimum value of daily minimum temperature), and mean annual rainfall. **b** Explanatory variables maps of the study region: projected future climate data during 2041–2060 based on SSP585 (Shared Socio-Economic Pathways) Tmax_mean (average of yearly maximum value of daily maximum temperature), Tmin_mean (average of yearly minimum value of daily minimum temperature), and mean annual rainfall. **c** Explanatory variables maps of the study region: Population density map of Sundarbans during 1991, 2001, and 2011. **d** Explanatory variables maps of the study region: land surface temperature (LST) of Indian Sundarbans during 1989, 2002, and 2019. **e** Explanatory variables maps of the study region: normalized difference vegetation index (NDVI) of Indian Sundarbans during 1989, 2002, and 2019. **f** Explanatory variables maps of the study region: soil salinity map estimated based on square value of canopy response salinity index (CRSISQR) of Indian Sundarbans during 1989, 2002, and 2019. **g** Explanatory variables maps of the study region: digital elevation map, distance to creeks, and distance to roads of Indian Sundarbans

**Fig. 10:**
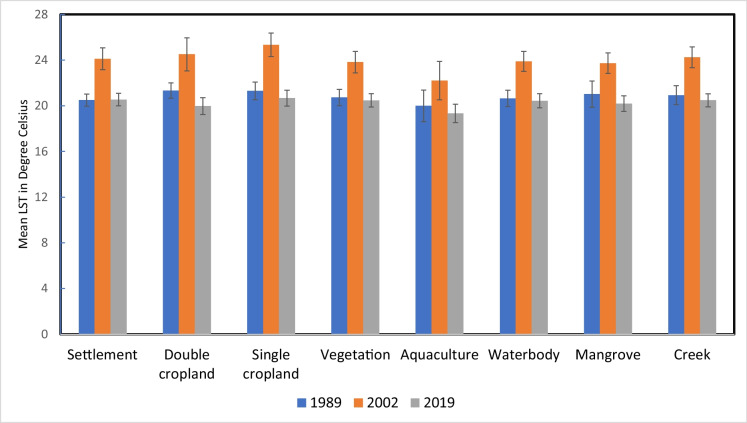
Land surface temperature over different LULC classes in 1989, 2002, and 2019

An extensive range of secondary data as explanatory spatial variables was used for predicting the study region’s LULC and soil salinity change (Figs. 9a–g). The land surface temperature (LST) for the study period was estimated using the thermal bands of the acquired imagery. For the years 1989, 2002, and 2019, the LST ranged from 16.56 to 26.67 °C, 19.28 to 31.24 °C, and 17.22 to 26.86 °C, respectively. The maximum and minimum LST values increased from 1989 to 2002 but decreased again during 2019. The mean LST distribution across various LULC classes for the years 1989, 2002, and 2019 was estimated using the zonal statistics in ArcGIS (Fig. 10). The lowest LST was consistently observed in aquaculture areas, while the highest temperatures were recorded in single-cropped areas for the years 2002 and 2019, which were fallow during the acquisition of imagery, and in double-cropped areas for the year 1989.

Our study utilized census data from 1991, 2001, and 2011, during which the population of the Indian Sundarbans increased from 3.1 million to 4.4 million. In the same period, the average population density rose from 760 to 1071 people per square kilometer. Population density influences the LULC, as its explosion leads to more settlement areas and urbanization of the region. According to the DEM of the region, the Indian Sundarbans comprise around 56% of the land, with an elevation of less than 5 m above mean sea level (MSL), whereas certain areas are even below the MSL. The road network influences the settlement area, and a buffer map has been generated for proximity to the road. Similarly, we created parallel buffers at distances of 1 km, 1–3 km, 3–5 km, and more than 5 km from rivers, creeks, and the sea to identify low-lying areas and those closer to the sea.

Historical and projected minimum, maximum temperature and annual rainfall data were used for our study. Historical minimum, maximum temperature, and rainfall varied from 8.66 to 8.73 °C, 41.49 to 41.66 °C and 1569 to 1574 mm, respectively, whereas the projected values as per SSP585 during 2041–2060 were 10.04–10.55 °C, 42.91–43.80 °C, and 1786–1860 mm, respectively. The projected minimum and maximum temperature will increase between 1.31–1.88 °C and 1.42–2.14 °C, respectively, and the mean annual rainfall may increase to 212–292 mm in the region. Above 40 °C maximum temperature is quite rare in Sundarbans, but the meteorological observatory located at ICAR-CSSRI, RRS, Canning Town, within the Indian part of Sundarbans, recorded 7 years between 2010 and 2014 where the maximum temperature at least once in a year was above 40 °C. During 2024, the observatory recorded a maximum of 43.6 °C in a day.

The soil salinity map was delineated using CRSISQR of the satellite imagery and verified with ground truth soil salinity (ECe) values. The overall classification accuracy of 0.84 with a kappa coefficient of 0.76 could be achieved for the CRSISQR map of 2019. The salinity map for 1989 and 2002 was generated based on CRSISQR of the respective year’s satellite imagery. A Spearman correlation matrix was constructed between the explanatory raster variables, one set with historical climate data and another set with projected future climate data (Tables 7 and 8). The LST and CRSISQR show a negative relationship, whereas NDVI and CRSISQR are positively related. Since CRSISQR and soil salinity are inversely related, higher CRSISQR values indicate lower soil salinity, which in turn supports greater vegetation growth, as reflected by higher NDVI values. Tmax_mean and CRSISQR are also negatively related, suggesting that higher temperatures contribute to increased soil salinity.

**Table 7:** Spearman’s correlation coefficients between historical climate data (1985–2020) and other explanatory raster variables

	CRSISQR 1989	CRSISQR 2002	Precipitation historical	Tmax_mean historical	Tmin_mean historical	DEM	Distance to road	Distance to creeks	LST 1989	LST 2002	NDVI 1989	NDVI 2002	Population 1991	Population 2001
CRSISQR 1989	**1**													
CRSISQR 2002	**0.57**	**1**												
Precipitation historical	**−0.20**	**−0.23**	**1**											
Tmax_mean historical	**−0.15**	0.06	**0.62**	**1**										
Tmin_mean historical	**−0.19**	**−0.18**	**0.87**	**0.26**	**1**									
DEM	0.01	**0.09**	**−0.10**	0.03	**−0.15**	**1**								
Distance to Road	0.05	**0.27**	**−0.38**	**−0.35**	**−0.22**	**0.08**	**1**							
Distance to Creeks	**−0.10**	**−0.26**	**0.42**	**0.35**	**0.24**	**−**0.06	**−0.49**	**1**						
LST 1989	**−0.12**	**−0.59**	**−0.16**	0.05	**−0.24**	**−0.17**	**−0.40**	**0.10**	**1**					
LST 2002	**−0.19**	**−0.72**	−0.01	**0.31**	**−0.16**	**−0.16**	**−0.49**	**0.19**	**0.72**	**1**				
NDVI 1989	**0.39**	**0.46**	**−0.39**	**−0.25**	**−0.32**	**0.33**	**0.59**	**−0.34**	**−0.36**	**−0.37**	**1**			
NDVI 2002	**0.13**	**0.36**	**−0.22**	**−0.12**	**−0.18**	**0.39**	**0.44**	**−0.20**	**−0.39**	**−0.32**	**0.67**	**1**		
Population 1991	**−0.10**	**−0.33**	**0.56**	**0.43**	**0.37**	**−0.13**	**−0.68**	**0.59**	**0.27**	**0.41**	**−0.61**	**−**0.42	**1**	
Population 2001	**−0.12**	**−0.34**	**0.61**	**0.49**	**0.42**	**−0.13**	**−0.69**	**0.61**	**0.26**	**0.42**	**−0.62**	**−**0.43	**0.95**	**1**

Values in bold have significance level at *p* = 0.05

**Table 8:** Spearman’s Correlation coefficients between projected climate data (2041–2060) and other explanatory raster variables

	CRSISQR 1989	CRSISQR 2019	Precipitation 2041–2060	Tmax_mean 2041–2060	Tmin_mean 2041–2060	DEM	Distance to road	Distance to creeks	LST 1989	LST 2019	NDVI 1989	NDVI 2019	Population 1991	Population 2011
CRSISQR 1989	**1**													
CRSISQR 2019	**0.24**	**1**												
Precipitation 2041–2060	**0.20**	**0.41**	**1**											
Tmax_mean 2041–2060	**−0.20**	**−0.41**	**−0.98**	**1**										
Tmin_mean 2041–2060	**0.20**	**0.37**	**0.96**	**−0.98**	**1**									
DEM	0.01	**0.32**	**0.11**	**−0.12**	**0.09**	**1**								
Distance to road	0.05	**0.40**	**0.40**	**−0.40**	**0.35**	**0.08**	**1**							
Distance to creeks	**−0.10**	**−0.34**	**−0.44**	**0.43**	**−0.40**	−0.06	**−0.49**	**1**						
LST 1989	**−0.38**	**−0.39**	**0.13**	**−0.12**	**0.18**	**−0.17**	**−0.40**	**−0.10**	**1**					
LST 2019	**−0.13**	**−0.09**	**0.39**	**−0.39**	**0.37**	**0.09**	**0.23**	**−0.33**	**0.21**	**1**				
NDVI 1989	**0.39**	**0.62**	**0.42**	**−0.42**	**0.37**	**0.33**	**0.59**	**−0.34**	**−0.69**	**−0.57**	**1**			
NDVI 2019	**0.17**	**0.76**	**0.43**	**−0.44**	**0.39**	**0.43**	**0.55**	**−0.34**	**−0.33**	**−0.31**	**0.74**	**1**		
Population 1991	**−0.10**	**−0.51**	**−0.60**	**0.59**	**−0.54**	**−0.13**	**−0.68**	**0.59**	**0.27**	**−0.32**	**−0.61**	**−0.63**	**1**	
Population 2011	**−0.13**	**−0.53**	**−0.64**	**0.64**	**−0.58**	**−0.13**	**−0.70**	**0.63**	**0.27**	**−0.33**	**−0.63**	**−0.63**	**0.94**	**1**

Values in bold have significance level at *p* = 0.05

Precipitation and CRSISQR exhibit a positive relationship under projected climate conditions but a negative relationship under historical climate conditions. In this study, we used mean annual precipitation rather than its temporal distribution. Higher precipitation generally promotes leaching of salts, thereby reducing soil salinity. However, excessive precipitation can sometimes lead to saline water inundation, resulting in increased soil salinity in the region. As no strong positive or negative correlation was reported between variables (except with Tmin_mean and precipitation and among population density; for all other variables, it was < 0.8), a total of 12 variables were taken as explanatory for predicting LULC and 9 variables for salinity prediction.

### Validation of the predicted LULC and salinity scenarios

The CA-ANN model of the QGIS-MOLUSCE Plugin software was employed to predict the LULC and salinity for the year 2015 and to compare it with the actual LULC and soil salinity maps of 2015 to validate the model’s reliability. The Cramer’s V values indicated that the selected variables were well-suited for transition potential modelling, with statistically significant associations at *p* = 0.05 (Table 9). The value of different kappa parameters showed a reasonably accepted level of accuracy (Table 10).

**Table 9:** Independent environmental variables and their Cramer’s V value for LULC and soil salinity projection/simulation

Independent environmental variables	Cramer’s V	
	Projection/simulation of LULC	Projection/simulation of soil salinity
Tmax_mean	0.201	0.115
Tmin_mean	0.230	0.120
Precipitation	0.270	0.125
Digital elevation model (DEM)	0.121	0.083
Land surface temperature (LST) 1989		0.084
Land surface temperature (LST) 2002		0.086
Distance from road	0.405	
Distance to creek	0.307	0.154
NDVI 1989	0.377	0.227
NDVI 2002	0.417	0.161
CRSISQR 1989	0.205	
CRSISQR 2002	0.419	
Population density 1991	0.422	
Population density 2001	0.417	

All the Cramer’s *V* values are significant at *p* = 0.05

**Table 10:** Validation of the LULC and soil salinity predicted maps using CA-ANN model for the year 2015

Prediction year	CA-ANN model validation using QGIS-MOLUSCE plugin			
	% Correctness	Overall kappa value	Kappa location	Kappa histogram
LULC 2015	73.08	0.63	0.68	0.93
Salinity 2015	86.3	0.57	0.82	0.69

The AUC values for prediction of LULC and soil salinity were 0.81 and 0.89, respectively, indicating that the transition models are sufficiently robust to project future land use and salinity patterns. Tables 11 and 12 show the actual and model output maps for the year 2015, as well as the projected scenario map for the year 2049 for LULC and soil salinity, respectively. The relative proportion of area under different LULC and salinity classes was the same for both actual and model predicted outputs.

**Table 11:** Actual and model output of LULC in 2015 as well as projected LULC during 2049

Classes	2015		Model prediction 2015		Projection 2049	
	Actual area (km^2^)	%	Projected area (km^2^)	%	Projected area (km^2^)	%
Settlement	181.7	3.1	164.1	2.8	243.1	4.2
Single cropland	2305.5	39.4	2339.1	40.0	2194.6	37.6
Double cropland	455.8	7.8	472.6	8.1	463.2	7.9
Vegetation	426.9	7.3	419.6	7.2	380.1	6.5
Waterbody	40.1	0.7	33.2	0.6	24.5	0.4
Creeks	98.9	1.7	103.6	1.8	54.7	0.9
Aquaculture	460.5	7.9	420.1	7.2	605.5	10.4
Mangrove	1883.6	32.2	1888.6	32.3	1875.3	32.1

**Table 12:** Actual and model output of salinity area during 2015, projected salinity area for the year 2049, actual salinity area in arable lands for 1989 and 2019, and comparison with projected area for the year 2049

Salinity classes	Year 2015		Model projection 2015		Projection 2049		1989*	2019*	2049*
	Actual area (km^2^)	%	Projected area (km^2^)	%	Projected area (km^2^)	%	Actual area (km^2^)	Actual area (km^2^)	Projected area (km^2^
Saline	3932.0	67.3	4597.1	78.7	3923.0	67.2	2246.2	2669.0	3232.6
Slightly-saline	1177.1	20.2	1086.5	18.6	876.8	15.0	816.6	320.3	48.1
Non-saline	731.8	12.5	157.3	2.7	1041.1	17.8	344.7	248.5	111.8
							% salinity area 89.9	% salinity area 92.3	% salinity area 96.7

*Salinity area was calculated within arable land of single cropland, double cropland, and aquaculture area

### Predicted LULC and salinity for 2049

**Fig. 11:**
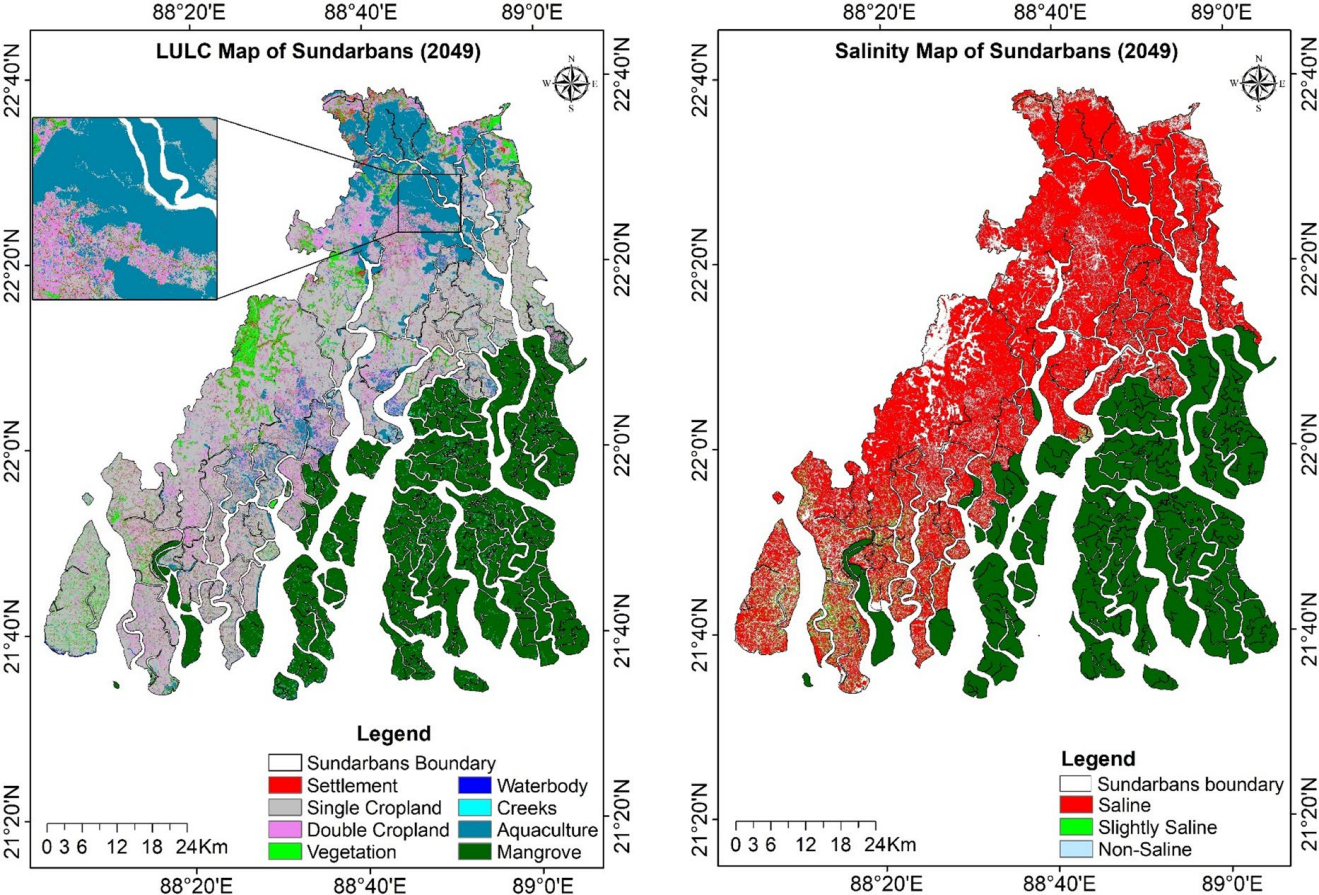
Projected LULC and salinity map of Indian Sundarbans for 2049 based on CA-ANN model

Classified LULC maps of the years 1989 and 2019 were used to simulate the projected LULC scenario for the year 2049. The future map indicated a likely expansion of settlement and aquaculture areas, primarily with the replacement of single-cropped areas of agricultural lands. The model also projected a notable decline in vegetation cover by 2049 (Fig. 11). Compared to the LULC of 2019, the simulated LULC of 2049 would face an increase of 32% and 30% in settlement and aquaculture areas and a decrease of 33.3%, 12.6%, 6.6%, and 4% in creeks, vegetation, double-cropped, and single-cropped areas, respectively. The mangrove area will remain stable with a transition value of 0.99 (Supplementary Table 8). Around 45% of the settlement area remained stable during 2019–2049, while single-cropped and vegetation areas would contribute 23% and 21% of their area. The transition matrix shows around 74.4% of the aquaculture areas will remain stable from 2019 to 2049, whereas 14% of its area will be contributed by the conversion of single-cropped land.

The soil salinity was estimated based on the CRSISQR dataset, and on the basis of the soil salinity map of 1989 and 2019, the salinity scenario of 2049 was predicted for the study area (Fig. 11, Table 12). The simulated scenario exposed higher salinity in arable areas of single and double cropland and aquaculture during 2049. The transition matrix between 2019 and 2049 showed that around 86% of the saline area will remain stable, whereas 5% and 9% of the area will be contributed by non-saline and slightly saline areas during the year 2049 (Supplementary Table 9).

## Discussion

### Erosion and accretion of Sundarbans

A notable characteristic of the Sundarbans coastline is the decreasing accretion rate relative to erosion over successive periods. This reduction in accretion is likely due to sediment deficits in the delta, primarily caused by anthropogenic interventions including dams (Paul et al., 2023; Syvitski et al., 2009). While erosion rates have remained relatively stable, accretion rates have decreased from 1973 to 2015. The erosion and accretion rates during 2019–2022 were comparatively higher than those during 2015–2019, perhaps due to four devastating cyclones that affected the area during 2019–2022. Mishra et al. (2021) reported severe erosion along the shoreline due to Cyclone *Amphan* during 2020 using geospatial technology. Roy and Ghosh (2024) used MODIS satellite images to assess the impact of cyclone *Yaas* and reported substantial variations in the land use land cover of the islands in the Sundarbans. Erosion has primarily affected the southern part of the Sundarbans coastline, likely reflecting the ongoing impact of SLR across the region. This area is exposed to southwest-directed tidal waves (Poterma et al., 1999). The combined forces of waves and tides are stronger in the southern direction, which may be the probable cause of the higher erosion rates in this area than in others.

Overall, the coastline of Sundarbans experienced a loss of 36 km^2^ of land at an annual rate of 0.74 km^2^ yr^−1^ during the 49 years of this study (Table 3). However, what is striking is that out of this net land loss, maximum erosion occurs mainly in the southern edge of Sundarbans covered under mangroves, which act as the main defence against frequent cyclonic storms. In contrast, accretion occurred in the creeks, which act as the main drainage line from the north. The lack of upland freshwater discharge has turned these creeks into flood-dominated estuaries where sediment-laden waters from the Bay of Bengal travel far inland during high tides, depositing sediment in the channels. Sedimentation in those creeks leads to frequent flooding/water logging in nearby areas, including Kolkata. The study reveals that the entire southern edge of the Indian Sundarbans is undergoing coastal retreat. However, this loss is not uniform across the region, likely due to localised factors such as variations in sedimentation rates, sediment compaction, and varying intensities of wave action.

The dynamic nature of the shoreline of Sundarbans is due to the development activities along the coast and in river catchment areas which have disrupted sediment transport equilibrium, leading to undesirable erosion and accretion patterns. Additionally, rising sea levels and local subsidence have further altered coastal processes. The river discharges from the GBM system significantly affect the Sundarbans coast which has been disrupted since the Farakka Barrage was built in 1975. Unlike other islands, Nayachar increased by 52% in area. Nayachar’s 52% area increase is explained by the powerful hydrological process of estuarine accretion, primarily driven by high sediment loads from the Hooghly River. This natural process has been enhanced and influenced by both natural factors and human interventions in the dynamic environment of the Ganga–Brahmaputra delta system (Islam et al., 2025).

Overall, approximately 15% of the coastline in India is experiencing erosion, 14% is undergoing accretion, and 70% remains stable, whereas in West Bengal, where the Sundarbans lies, the highest erosion rates are observed, with 36% of its coastline affected (Ratheesh et al., 2023). Factors such as wave exposure, high tidal currents, sediment scarcity, and low-lying characteristics make the West Bengal coastline especially vulnerable to erosion. Hajra et al. (2023) also reported that the islands of Sundarbans are severely eroded due to climate change-induced SLR, tropical cyclones, and tidal surges.

### Land use and salinity dynamics and future transition

The land dynamics analysis from 1973 to 2022 revealed that the maximum reduction in area occurred in single-cropped land, which declined by 13.35%. In contrast, the highest gain was observed in aquaculture, which increased by 5.02%, followed by vegetation area (3.61%) and double-cropped area (3.48%) (Table 6). A remarkable increase of seven times in settlement area was visible to meet the population growth of the region during the study period. However, the changes across land use/land cover categories were not uniform over time. Notably, the transition from single-cropped land to double-cropped land and aquaculture occurred faster during the period from 1989 to2002 compared to other study periods.

The trend of change in LULC during the years 1973–1989–2002–2011–2015–2019–2022 was characterized by consistent and significant expansion of aquaculture and double-cropped area, mainly through the sacrifice of mono-cropped area. A similar shift for aquaculture area was reported by DasGupta et al. (2019) in the Indian Sundarbans delta and noticed in Bangladesh’s coastal part more rapidly, where the shrimp farming area increased from 38.5 to 172.1 km^2^ from 1989 to 2010 (Islam et al., 2016). The brackish water aquaculture, particularly shrimp farming, is one of the most remunerative farming practices in the region. Shrimps have developed a strong industry in Asia, and India has become the world’s top shrimp producer. Also, there is a lot of demand for shrimp and other brackish water fishes in the nearby Kolkata market. An uneven shift was also observed due to urban expansion, most notably in the settlement area, which increased from 27.3 to 191.5 km^2^ at an average annual rate of 3.4 km^2^. Mono-cropped area decreased linearly from 3140.3 km^2^ to 2339.1 km^2^ with an annual reduction rate of 16.3 km^2^, whereas double-cropped and aquaculture areas expanded at a rate of 4.5 and 6.5 km^2^ per year.

A total of 12 and 9 environmental variables were considered for LULC and soil salinity prediction, respectively, based on Cramer’s *V* value (Table 9). The NDVI was positively correlated with CRSISQR, which indicated that vegetation is inversely related to soil salinity (Tables 7 and 8). NDVI was also inversely related to population density as vegetation and agricultural activity were concentrated in relatively lesser populated areas. Although the region is mostly flat, the DEM showed a positive correlation with NDVI, suggesting that agricultural activities were more concentrated in highland areas away from the coast. The population density is one of the important anthropogenic features influencing the spatial distribution of LULC and is considered one of the explanatory variables for LULC change prediction. Islam et al. (2023) also used population as one of the drivers for quantifying forest land use changes using CA-ANN in Bangladesh. The overall kappa value (Table 10) for validation of LULC (0.63) and soil salinity (0.57) indicates that the CA-ANN model performed reasonably accurately considering the dynamic nature of the Sundarbans as well as the complexity of salinity prediction. High AUC value (0.81 for LULC and 0.89 for soil salinity) in our study also supports this. Comparable machine learning-based studies using MOLUSCE reported kappa values of 0.48, 0.54, and 0.63 by Muhammad et al (2022), Mkrtchian and Syidzinska (2017), and Rahman et al. (2017), respectively.

The future LULC scenario, as predicted (Table 11 and Fig. 11), indicates approximately a 13% reduction in vegetation area by 2049 compared to 2019. The settlement area in the Sundarbans region is heavily covered with vegetation. The inhabitants of the coastal zone used to adapt and manage the frequent storms and cyclones, usually by building their homes on raised floors, keeping height low, and being surrounded by highly protective windbreaks plantation. An increase in settlement areas and a reduction in vegetation cover suggest an increase in urbanization which may cause adverse effects on urban health, ecosystem services, and thermal characteristics. As the population continues to grow, the vegetation surrounding households may be converted into residential areas to accommodate this increase. If unplanned urban expansion continues, it will likely lead to significant environmental and economic challenges in the study area (Kafy et al., 2021). The rising urban footprints coupled with decreased vegetation coverage will exacerbate the issue of temperature increases in the region (Xiang et al., 2024). Moreover, the increase in settlement areas and decrease in vegetation cover intensify evaporation rates, leading to the accumulation of salt within the soil profile and consequently contributing to increased soil salinity (Mondal et al., 2025).

The aquaculture area has been growing steadily since 1973 and will continue to grow in the future because of its comparative advantage in remuneration over rice cultivation. The shift towards brackish water aquaculture, especially shrimp farming, has an adverse effect on the socio-environmental situation not only in the Sundarbans region but also in other coastal states of India. These impacts include the loss of croplands, degradation of mangroves in fringe areas, loss of biodiversity, degradation of soil and water quality, disease outbreaks, and economic displacement. Furthermore, brackish water farming reduces rice productivity in nearby fields by increasing the salinity and acidity of the soil of varying degrees, which causes soil degradation in the regions. Farmers sometimes even use brackish groundwater to irrigate their ponds not only in the coastal region but also in the inland saline areas of India, which can have a serious environmental impact on nearby agricultural lands. The challenges associated with brackish water farming, including its competition with rice cultivation, ecological impacts, and social disputes, pose significant hurdles for decision-making and policy development. As a result, it is crucial to develop a comprehensive, environmentally friendly, and socially acceptable management strategy for brackish water farming.

One of the most striking findings of our study is that approximately 99% of the mangrove area is projected to remain stable, largely due to effective conservation and afforestation programs. This contrasts with many land dynamic studies in India and China, which have reported a decrease in forest cover resulting from increased urbanization and cropland expansion driven by anthropogenic pressures (Kamaraj and Rangarajan, 2022; Muhammad et al., 2022). For example, following the *Amphan* cyclone in May 2020, the Government of West Bengal, India, planted 123.77 million mangrove saplings across 11 blocks of the Sundarbans Biosphere Reserve, actively involving local communities in the restoration efforts (Banerjee et al., 2023).

Around 38% of the area will remain single-cropped even in 2049. Agriculture in coastal areas is inherently risk-prone because of variable climate and environments (water tables, soil type, and local salinity dynamics). Environmental changes have led to increased uncertainty in agriculture, affecting cropping patterns due to unpredictable precipitation and the consequences of floods, droughts, salinity, cyclones, and hailstorms. Therefore, strategic plans need to adopt climate-smart and resilient agricultural technologies for sustainable crop production under a rapidly changing climate to make the region self-sufficient in food security. The LULC of 2049 also predicts a 33.3% reduction in the creeks area. The area experiences rapid morphological changes due to the dynamic movement of water and sediment, resulting in varying rates of erosion and accretion over time. More accretion than erosion is responsible for the reduction of the creeks area towards the landward side of the study region. Although the double-cropped area increased steadily from 1973 to 2022, the model predicts a 7% reduction in its area in 2049 compared to 2019. While the CA-ANN model is generally effective in forecasting land cover changes under the assumption of consistent historical patterns, it may fall short in providing precise spatial predictions. As noted by Shatnawi and Abu Qdais (2019), the model lacks standardized guidelines for weighting each input parameter. However, achieving 100% accuracy in forecasting dynamic phenomena like LULC is challenging due to the influence of human activities.

In our study, CRSISQR was used to delineate the soil salinity areas. Mandal et al. (2023, 2025) delineated the coastal soil salinity map of India for the Ganges delta region within coastal Bangladesh and West Bengal, India, and verified it with ground soil salinity values. The soil salinity is negatively related to CRSISQR, and the index has been extensively used for regional scale salinity mapping (Ramos et al., 2020; Scudiero et al., 2015).

The LST was considered a one of the explanatory variables for estimating soil salinity as it influenced soil drying, leading to increased capillary rise of shallow saline groundwater and higher salt accumulation on the soil surface. The acquisition period of imagery plays a role in the variation of LST in different years of the study. Different surface materials in the region exhibit varying emissivity values (Dar et al., 2019; Neteler, 2010). The increase in land surface temperature (LST) may be attributed to factors such as unplanned urbanization, the impact of climate change, and reductions in vegetation and surface waterbodies within the study area (Alamgir et al., 2020; Xiang et al., 2024). Analysis revealed a negative correlation between LST and NDVI, indicating that areas with higher vegetation cover tend to have lower surface temperatures (Tables 7 and 8). Similarly, a negative relationship was observed between LST and CRSISQR, suggesting a linkage between elevated surface temperatures and salinity stress in vegetation. Kafy et al. (2021) also reported a negative relationship between LST and NDVI while modeling the relationship between LULC and LST in Dhaka, Bangladesh. Mondal et al. (2025) also reported a significant positive correlation between average soil salinity and temperature. Geospatial factors such as topography, temperature, and rainfall distribution and river connectivity play crucial roles in shaping salinity gradients in low-lying coastal zones. Though the sea level rise is one of the highest in the Sundarbans delta, the parameter was not considered for LULC and salinity prediction due to the lack of a sufficient spatially variable database of SLR. Instead of SLR, we used proximity to creeks map as one of the explanatory variables for LULC and salinity prediction.

The simulated scenario predicted 96.7% of arable lands under single crop, double crop, and aquaculture would be saline by 2049 from 92.3% of arable lands area in 2019 (Table 12). Climate projections for 2041–2060 anticipated a rise in maximum and minimum temperature and increased annual rainfall, which could impact salt removal from surface soils. Conversely, expanding aquaculture areas will lead to greater saltwater intrusion and higher soil salinity.

Considering the regions’ land dynamics, the number of measures has been suggested for the long-term sustainability of the vulnerable Sundarbans region. Land gain areas due to accretion can be targeted for planting salt-tolerant mangrove species instead of the expansion of settlements, while areas experiencing land loss can be addressed through the selective construction of concrete dikes combined with mangrove planting. Embankments are crucial for the existence of human settlements in the islands, and hard engineering structures along with mangrove colonization are recommended to protect against embankment erosion. Strategic dredging practiced since the British era is still effective for improving navigation of rivers. The brackish water system is devoid of any vegetation because of extremely high salinity. No other crop can grow in this saline environment except native mangrove. An integrated mangrove aquaculture system in brackish water farms can improve the microclimate, sequester blue carbon, and support the region’s climate change adaptation and mitigation. A viable strategy for sustainable coastal agriculture involves the intensification of agri-aquaculture systems through land shaping techniques that allow the capture of surplus runoff. This stored water can then be used for supplemental irrigation during critical crop growth stages. Coastal salinity can be managed through appropriate water management and effective mulching. Additionally, implementing contingency planning, real-time weather forecasting with agro-advisory services, and crop insurance can further strengthen the resilience of the agricultural system.

### Limitation and future research directions

In this study, LULC change dynamics and soil salinity in the deltaic Sundarbans were evaluated, and CA-ANN machine learning tools were used for future prediction of LULC and soil salinity, whereas this study also has a few limitations that should be considered while interpreting the results.

- Firstly, key environmental factors such as seawater intrusion, groundwater salinity, and sea level rise, which significantly affect soil salinity, were not included in the analysis. These elements could potentially exacerbate the salinity problem, and future research should aim to incorporate these factors for a more comprehensive understanding of salinity dynamics in coastal regions.
- Secondly, the classification of land use types, such as mangroves or creeks and tidal flats, as single classes may have overlooked finer details like species composition within mangroves and landform variations, which could reveal more detailed changes. A more detailed and specific classification system could help identify these finer-scale changes and their impact on salinity and land use patterns.
- Third is the limited consideration of socioeconomic factors such as policy shifts and market dynamics. While the primary focus was on natural drivers of climate change impacts, the interaction between these natural factors and socioeconomic conditions plays a critical role in shaping real-world outcomes. Future research should aim to incorporate these socioeconomic dimensions to provide a more comprehensive and realistic assessment of climate change impacts.
- Additionally, the CA-ANN algorithm, while effective for simulating future LULC patterns, can produce inconsistent results due to its lack of transparency in handling input parameters and assigning weights. Robust sensitivity analyses and uncertainty quantification methods are essential to assess the impact of data variability and model assumptions on prediction outcomes. Models should be flexible enough to incorporate stakeholder knowledge and expert inputs relevance to real-world conditions.
- Finally, the reliance on low resolution satellite data limits the ability to predict salinity and LULC changes at finer scales. Future research should focus on integrating multi-resolution satellite data and advanced remote sensing techniques to improve the precision and accuracy of predictions, particularly in regions where small-scale changes in land use and salinity are important.

## Conclusion

The study assessed erosion, accretion, and LULC dynamics and predicted future LULC and surface soil salinity in the climatically vulnerable Indian Sundarbans. This was accomplished using climatic variables (temperature and rainfall), NDVI, CRSISQR, DEM, LST, and spatial variables such as distance to roads, distance to rivers, and population density, through the application of CA-ANN algorithms. Spanning a period of 49 years, the analysis revealed a significant transition of single-cropped areas to aquaculture and double-cropped land, while settlement areas expanded nearly sevenfold to accommodate population growth. Monitoring long-term LULC changes is essential for maintaining ecosystem integrity and ensuring environmental sustainability.

A notable finding of this study is the stability of the mangrove forest area over the 49 years period, attributed to effective protection measures, restrictions within reserved forest areas, and ongoing maintenance efforts. Detecting LULC changes, predicting future trends, and assessing soil salinity are critical for mitigating salinity’s adverse effects on agriculture and promoting sustainable development. The soil salinity indices help in identifying areas at risk, evaluating the impacts on agriculture, assessing mitigation strategies, and enhancing decision-making and community preparedness, thereby reducing economic repercussions. Although forecasting the dynamics of LULC and salinity with complete accuracy remains challenging due to the dominant influence of human activities, dynamic modelling serves as a useful tool for hypothesis formulation and informed decision-making to manage and conserve vital natural resources.

## Supplementary information

Below is the link to the electronic supplementary material.

Supplementary file 1 (DOCX 39.0 KB)

## Data Availability

The data are available upon request.

## Abbreviations

LULC: Land use land cover
CRSI: Canopy response salinity index
CRSISQR: Square value of canopy response salinity index
CA-ANN: Cellular automata-artificial neural network
MOLUSCE: Modules of land use change evaluation
SLR: Sea level rise
GBM: Ganga-Brahmaputra-Meghna
USGS: US Geological Survey
UTM: Universal Transverse Mercator
DN: Digital number
FCC: False color composite
NDVI: Normalized difference vegetation index
NIR: Near-infrared
ECe: Electrical conductivity of saturation paste extract
TM: Thematic mapper
ETM +: Enhanced thematic mapper plus
OLI: Operational land imager
TIRS: Thermal infrared sensor
FAO: Food and Agriculture Organization
DEM: Digital elevation model
dS m^−^^1^: Decisiemens per meter
Tmax_mean: Average of yearly maximum value of daily maximum temperature
Tmin_mean: Average of yearly minimum value of daily minimum temperature
ECMWF: European Centre for Medium-Range Weather Forecasts
IPCC: Intergovernmental Panel on Climate Change
SSP: Shared Socio-Economic Pathways
LST: Land surface temperature
TB: Brightness temperature
FV: Fractional vegetation
ROC: Receiver operating characteristic
AUC: Area under curve
MSL: Mean sea level

## References

[CR1] Alamgir, M., Khan, N., Shahid, S., Yaseen, Z. M., Dewan, A., Hassan, Q., & Rasheed, B. (2020). Evaluating severity–area–frequency (SAF) of seasonal droughts in Bangladesh under climate change scenarios. *Stochastic Environmental Research and Risk Assessment,**34*(2), 447–464.

[CR2] Banerjee, S., Ladd, C. J. T., Chanda, A., Shil, S., Ghosh, T., Large, A., & Balke, T. (2023). Commentary: Securing the sustainable future of tropical deltas through mangrove restoration: lessons from the Indian Sundarban. *One Earth, 6*, 190–194.

[CR3] Carlson, T. N., & Ripley, D. A. (1997). On the relation between NDVI, fractional vegetation cover, and leaf area index. *Remote Sensing of Environment,**62*(3), 241–252.

[CR4] Chander, G., Markham, B. L., & Helder, D. L. (2009). Summary of current radiometric calibration coefficients for Landsat MSS, TM, ETM+, and EO-1 ALI sensors. *Remote Sensing of Environment,**113*, 893–903.

[CR5] Dar, I., Qadir, J., & Shukla, A. (2019). Estimation of LST from multi-sensor thermal remote sensing data and evaluating the influence of sensor characteristics. *Annals of GIS,**25*(3), 263–281.

[CR6] DasGupta, R., Hashimoto, S., Okuro, T., & Basu, M. (2019). Scenario-based land change modelling in the Indian Sundarban delta: An exploratory analysis of plausible alternative regional futures. *Sustainability Science,**14*, 221–240. 10.1007/s11625-018-0642-6

[CR7] Day, J. W., Christian, R. R., Boesch, D. M., Y´a˜nez-Arancibia, A., Morris, J., Twilley, R. R., Naylor, L., Schaffner, L., & Stevenson, C. (2008). Consequences of climate change on the ecogeomorphology of coastal wetlands. *Estuaries and Coasts,**31*, 477–491. 10.1007/s12237-008-9047-6

[CR8] FAO (2020). Food and Agriculture Organization of the United Nations, Mapping of Salt-Affected Soils: Technical Manual, Rome, Italy.

[CR9] Ghosh, A., Schmidt, S., Fickert, T., & Nüsser, M. (2015). The Indian Sundarban mangrove forests: History, utilization, conservation strategies and local perception. *Diversity,**7*, 149–169. 10.3390/d7020149

[CR10] Giri, C., Pengra, B., Zhu, Z., Singh, A., & Tieszen, L. (2007). Monitoring mangrove forest dynamics of the Sundarbans in Bangladesh and India using multi-temporal satellite data from 1973–2000. *Estuarine, Coastal and Shelf Science,**73*(1–2), 91–100.

[CR11] Hajra, R., Mitra, R., & Ghosh, T. (2023). Sustainability assessment of Indian Sundarban delta islands using DPSIR framework in the context of natural hazards. *Natural Hazards Research,**3*, 76–88.

[CR12] Huang, W., Hashimoto, S., Yoshida, T., Saito, O., & Meraj, G. (2024). Understanding Japan’s land-use dynamics between 1987 and 2050 using land accounting and scenario analysis. *Sustainability Science,**19*, 1561–1577. 10.1007/s11625-024-01517-2

[CR13] Islam, A., Khatun, J., Sarkar, B., Mohinuddin, S., Sengupta, S., Pal, S. C., & Islam, A. R. M. T. (2025). Evaluating island evolution and shoreline dynamics for better community resilience: An example from a tropical island in the face of the Bay of Bengal. *Regional Studies in Marine Science,**90*, Article 104483. 10.1016/j.rsma.2025.104483

[CR14] Islam, M. Y., Nasher, N. M. R., Krim, K. H. R., & Rashid, K. J. (2023). Quantifying forest land-use changes using remote-sensing and CA-ANN model of Madhupur sal forest, Bangladesh. *Heliyon,**9*, Article e15617. 10.1016/j.heliyon.2023.e15617 37159710 10.1016/j.heliyon.2023.e15617 PMC10163617

[CR15] Islam, R. M., Miah, M. G., & Inoue, Y. (2016). Analysis of land use and land cover changes in the coastal area of Bangladesh using landsat imagery. *Land Degradation & Development,**27*, 899–909.

[CR16] Kafy, A. A., Bakshi, A., Saha, M., Faisal, A. A., Almulhim, A. I., Rahaman, Z. A., & Mohammad, P. (2023). Assessment and prediction of index based agricultural drought vulnerability using machine learning algorithms. *Science of the Total Environment,**867*, Article 161394. 10.1016/j.scitotenv.2023.161394 36634773 10.1016/j.scitotenv.2023.161394

[CR17] Kafy, A. A., Dey, N. N., Rakib, A. A., Rahaman, Z. A., Nasher, N. M. R., & Bhatt, A. (2021). Modeling the relationship between land use/land cover and land surface temperature in Dhaka, Bangladesh using CA-ANN algorithm. *Environmental Challenges,**4*, Article 100190.

[CR18] Kamaraj, M., & Rangarajan, S. (2022). Predicting the future land use and land cover changes for Bhavani basin, Tamil Nadu, India, using QGIS MOLUSCE plugin. *Environmental Science and Pollution Research,**29*, 86337–86348. 10.1007/s11356-021-17904-6 35112256 10.1007/s11356-021-17904-6

[CR19] Kamruzzaman, M., Shahid, S., Islam, A. R. M. T., Hwang, S., Cho, J., & Zaman, M. A. U. (2021). Comparison of CMIP6 and CMIP5 model performance in simulating historical precipitation and temperature in Bangladesh: A preliminary study. *Theoretical and Applied Climatology,**145*, 1385–1406.

[CR20] Karim, F., Yu, Y., Kamruzzaman, M., Mandal, U. K., Zahan, T., Paul, P., & Mainuddin, M. (2024). Assessing changes in climate extremes using CMIP6 and its implications for agriculture in the Ganges Delta. *Journal of the Indian Society of Coastal Agricultural Research,**42*(1), 33–49. 10.54894/JISCAR.42.1.2024.147069

[CR21] Khalid, W., Shamim, S. K., & Ahmad, A. (2024). Synergistic approach for land use and land cover dynamics prediction in Uttarakhand using cellular automata and artificial neural network. *Geomatica,**76*, Article 100017. 10.1016/j.geomat.2024.100017

[CR22] Kumar, P., Tiwari, P., Biswas, A., & Srivastava, P. K. (2024). Spatio-temporal assessment of soil salinization utilizing remote sensing derivatives, and prediction modeling: Implications for sustainable development. *Geoscience Frontiers,**15*, Article 101881.

[CR23] Lal, R. (2022). Coastal ecosystems of India and their management of enhance blue carbon storage. In: Transforming Coastal Zone for Sustainable Food and Income Security by Lama, T.D., Burman, D., Mandal, U.K., Sarangi, S.K., & Sen, H.S. (Eds.). Springer. 591–605.

[CR24] Lespinas, F., Ludwig, W., & Heussner, S. (2010). Impact of recent climate change on the hydrology of coastal Mediterranean rivers in Southern France. *Climatic Change,**99*, 425–456. 10.1007/s10584-009-9668-1

[CR25] Mandal, U. K., Burman, D., Bhardwaj, A. K., Nayak, D. B., Samui, A., Mullick, S., Mahanta, K. K., Lama, T. D., Maji, B., Mandal, S., Raut, S., & Sarangi, S. K. (2019). Waterlogging and coastal salinity management through land shaping and cropping intensification in climatically vulnerable Indian Sundarbans. *Agricultural Water Management,**216*, 12–26.

[CR26] Mandal, U. K., Karim, F., Yu, Y., Ghosh, A., Zahan, T., Mallick, S., Kamruzzaman, M., Chandra Paul, P. L., & Mainuddin, M. (2025). Assessing vulnerability and climate risk to agriculture for developing resilient farming strategies in the Ganges Delta. *Climate Risk Management,**47*, Article 100690. 10.1016/j.crm.2025.100690

[CR27] Mandal, U. K., Nayak, D. B., Ghosh, A., Bhardwaj, A. K., Lama, T. D., Mahajan, G. R., Das, B., Nagaraja, M. S., Vittal B.K., Rani, P. P., Mal, S., Samui, A., Mahanta, K. K., Mandal, S., Raut, S., & Burman, D. (2023). Delineation of saline soils in coastal India using satellite remote sensing. *Current Science,**125*(12), 1339–1353.

[CR28] Mandal, U. K., Nayak, D. B., Samui, A., Jana, A. K., Mullick, S., Lama, T. D., Bhardwaj, A. K., Mahanta, K. K., Mandal, S., Raut, S., Sarangi, S. K., & Burman, D. (2018). Trend of sea-level-rise in West Bengal coast. *Journal of the Indian Society of Coastal Agricultural Research,**36*(2), 64–73.

[CR29] Mishra, M., Acharyya, T., Santos, C. A. G., da Silva, R. M., Kar, D., Kamal, A. H. M., & Raulo, S. (2021). Geo-ecological impact assessment of severe cyclonic storm Amphan on Sundarban mangrove forest using geospatial technology. *Estuarine, Coastal and Shelf Science,**260*, Article 107486. 10.1016/j.ecss.2021.107486

[CR30] Mkrtchian, A., & Svidzinska, D. (2017). Quantifying landscape changes through land cover transition potential analysis and modeling (on the example of the Black Tisza river basin). Landscape and Landscape Ecology, In: Proceedings of the 17th International Symposium on Landscape Ecology, 27–29 May 2015, Nitra, Slovakia, Institute of Landscape Ecology, Slovak Academy of Sciences, Bratislava, p. 141.

[CR31] Mondal, I., Hossain, S. K. A., Das, A., Jose, F., Altuwaijri, H. A., & Juliev, M. (2025). Exploring ML- driven insights on the impact of rising soil salinity on Sundarbans mangrove ecosystems and ecological sustainability through nature- based solutions. *Land Degradation & Development,**0*, 1–23. 10.1002/ldr.70105

[CR32] Muhammad, R., Zhang, W., Abbas, Z., Guo, F., & Gwiazdzinski, L. (2022). Spatiotemporal change analysis and prediction of future land use and land cover changes using QGIS MOLUSCE plugin and remote sensing big data: A case study of Linyi, China. *Land,**11*(3), Article 419. 10.3390/land11030419

[CR33] Naeem, M., Zhang, Y., Tian, X., Miao, P., Li, C., Xu, Z., Wang, L., Mumtaz, F., Tang, Z., & He, S. (2025). Assessing and predicting Bojiang lake area and LULC changes from 2000 to 2045. *Journal of Hydrology: Regional Studies,**58*, Article 02216. 10.1016/j.ejrh.2025.102216

[CR34] Nelson, S. A., & Khorram, S. (2018). *Image processing and data analysis with ERDAS IMAGINE®* (p. 329). CRC Press, Taylor and Francis Group.

[CR35] Neteler, M. (2010). Estimating daily land surface temperatures in mountainous environments by reconstructed MODIS LST data. *Remote Sensing (Basel),**2*, 333–351.

[CR36] Paul, S., Das, C. S., & Chaudhuri, S. (2023). Erosion-accretion pattern in the tidal-rivers of Indian Sundarban in relation to estuarine hydrodynamics. *Continental Shelf Research,**252*, Article 104865. 10.1016/j.csr.2022.104865

[CR37] Poterma, J. T., Luther, M. E., & O’Brien, J. J. (1999). The seasonal circulation of the upper ocean in the Bay of Bengal. *Journal of Geophysical Research,**96*(C7), 12667–12683.

[CR38] Quader, M. A., Agrawal, S., & Kervyn, M. (2017). Multi-decadal land cover evolution in the Sundarban, the largest mangrove forest in the world. *Ocean & Coastal Management,**139*, 113–124. 10.1016/j.ocecoaman.2017.02.008

[CR39] Rahman, A. F., Dragoni, D., & El-Masri, B. (2011). Response of the Sundarbans coastline to sea level rise and decreased sediment flow: A remote sensing assessment. *Remote Sensing of Environment,**115*, 3121–3128.

[CR40] Rahman, M. T. U., Tabassum, F., Rasheduzzaman, M., Saba, H., Sarkar, L., Ferdous, J., Uddin, S. Z., & Islam, A. Z. (2017). Temporal dynamics of land use/land cover change and its prediction using CA-ANN model for southwestern coastal Bangladesh. *Environmental Monitoring and Assessment,**189*(11), Article 565. 10.1007/s10661-017-6272-0 29039035 10.1007/s10661-017-6272-0

[CR41] Ramos, T. B., Castanheira, N., Oliveira, A. R., Paz, A. M., Darouich, H., Simionesei, L., Farzamian, M., & Gonçalves, M. C. (2020). Soil salinity assessment using vegetation indices derived from Sentinel-2 multispectral data. application to Lezíria Grande, Portugal. *Agricultural Water Management, 241*, 106387. 10.1016/j.agwat.2020.106387

[CR42] Ratheesh, R., Rajput, P., Bhatti, H., Rajawat, A. S., & Ram, R. D. (2023). Quantification of shoreline changes along the entire Indian coast using Indian remote sensing satellite images of 2004–06 and 2014–16. *Current Science,**124*(5), 578–584.

[CR43] Rouse, J., Haas, R., Schell, J., & Deering, D. (1973). Monitoring vegetation systems in the Great Plains with ERTS. In Third ERTS Symposium, NASA SP-351, Washington DC, pp. 309–317; s11769–014–0693–2.

[CR44] Roy, S. S., & Ghosh, T. (2024). Local-level impacts of cyclone Yaas on the islands of the Indian Sundarbans delta. *Natural Hazards,**120*, 3995–4010. 10.1007/s11069-023-06304-3

[CR45] Saha, T. K., Haroon, S., Roshani, Rahaman, Rahaman, M. H., & Sharma, Y. (2024). Exploring the impact of land use/land cover changes on the dynamics of Deepor wetland (a Ramsar site) in Assam, India using geospatial techniques and machine learning models. *Modeling Earth System and Environment,**10*, 4043–4065. 10.1007/s40808-024-01999-0

[CR46] Scudiero, E., Skaggs, T. H., & Corwin, D. L. (2015). Regional scale soil salinity evaluation using Landsat 7, western San Joaquin Valley, California, USA. *Geoderma Regional,**2*, 82–90. 10.1016/j.geodrs.2014.10.004

[CR47] Shatnawi, N., & Abu Qdais, H. (2019). Mapping urban land surface temperature using remote sensing techniques and artificial neural network modelling. *International Journal of Remote Sensing,**40*, 1–16.

[CR48] Sobrino, J. A., Raissouni, N., & Li, Z. L. (2001). A comparative study of land surface emissivity retrieval from NOAA data. *Remote Sensing of Environment,**75*(2), 256–266.

[CR49] Sun, Y., Yin, X., & Mao, L. (2024). Landscape pattern prediction method based on ANN-CA-Markov coupling model. *Heliyon,**10*, Article e38012. 39386770 10.1016/j.heliyon.2024.e38012 PMC11462203

[CR50] Syvitski, J. P. M., Kettner, A. J., Overeem, I., Hutton, E. W. H., Hannon, M. T., Brakenridge, G. R., Day, J., Vörösmarty, C., Saito, Y., Giosan, L., & Nicholls, R. J. (2009). Sinking deltas due to human activities. *Nature Geoscience*. 10.1038/ngeo629

[CR51] Xiang, X., Zhai, Z., Fan, C., Ding, Y., Ye, L., & Li, J. (2024). Modelling future land use land cover changes and their impacts on urban heat island intensity in Guangzhou, China. *Journal of Environmental Management,**366*, Article 121787. 10.1016/j.jenvman.2024.121787 38981259 10.1016/j.jenvman.2024.121787

