# Supplementary material for: Land use change and soil salinization in the Sundarbans: a machine-learning based analysis of long-term transformation and future projections
Source: Environ Monit Assess. 2025 Nov 28;197(12):1380. doi: 10.1007/s10661-025-14829-2 (PMC12662945; doi:10.1007/s10661-025-14829-2)
Supplement: Supplementary file 1 — (DOCX 39.0 KB) [file 10661_2025_14829_MOESM1_ESM.docx]

**Supplementary information:**

**Supplementary Table 1** Transitional matrix of LULC categories between 1973 to 2022

| LULC Classes | | 2022 | | | | | | | |
| --- | --- | --- | --- | --- | --- | --- | --- | --- | --- |
|  |  | Aquaculture | Creeks | Double  Cropland | Single cropland | Mangrove | Settlement | Vegetation | Waterbody |
| 1973 | Aquaculture | 0.173 | 0.031 | 0.025 | 0.030 | 0.006 | 0.041 | 0.025 | 0.098 |
|  | Creeks | 0.025 | 0.137 | 0.023 | 0.028 | 0.017 | 0.042 | 0.029 | 0.109 |
|  | Double cropland | 0.163 | 0.002 | 0.050 | 0.071 | 0.000 | 0.038 | 0.025 | 0.067 |
|  | Single cropland | 0.570 | 0.078 | 0.840 | 0.826 | 0.011 | 0.720 | 0.741 | 0.573 |
|  | Mangrove | 0.029 | 0.741 | 0.003 | 0.007 | 0.964 | 0.004 | 0.005 | 0.105 |
|  | Settlement | 0.002 | 0.001 | 0.006 | 0.003 | 0.000 | 0.027 | 0.026 | 0.005 |
|  | Vegetation | 0.034 | 0.006 | 0.052 | 0.033 | 0.001 | 0.123 | 0.146 | 0.032 |
|  | Waterbody | 0.005 | 0.005 | 0.002 | 0.001 | 0.000 | 0.005 | 0.003 | 0.011 |

**Supplementary Table 2** Transitional matrix of LULC categories between 1973 to 1989

| LULC Classes | | 1989 | | | | | | | |
| --- | --- | --- | --- | --- | --- | --- | --- | --- | --- |
|  |  | Aquaculture | Creeks | Double  Cropland | Single cropland | Mangrove | Settlement | Vegetation | Waterbody |
| 1973 | Aquaculture | 0.33 | 0.02 | 0.02 | 0.022 | 0.003 | 0.0225 | 0.02 | 0.0795 |
|  | Creeks | 0.04 | 0.16 | 0.02 | 0.024 | 0.017 | 0.0447 | 0.03 | 0.0939 |
|  | Double cropland | 0.11 | 0.01 | 0.09 | 0.073 | 0.001 | 0.0258 | 0.02 | 0.0441 |
|  | Single cropland | 0.46 | 0.18 | 0.81 | 0.836 | 0.010 | 0.6311 | 0.66 | 0.6655 |
|  | Mangrove | 0.02 | 0.61 | 0.00 | 0.011 | 0.966 | 0.0035 | 0.00 | 0.0085 |
|  | Settlement | 0.00 | 0.00 | 0.00 | 0.002 | 0.001 | 0.0509 | 0.04 | 0.0154 |
|  | Vegetation | 0.03 | 0.02 | 0.06 | 0.030 | 0.002 | 0.2138 | 0.22 | 0.0842 |
|  | Waterbody | 0.01 | 0.00 | 0.00 | 0.002 | 0.000 | 0.0077 | 0.00 | 0.0090 |

**Supplementary Table 3** Transitional matrix of LULC categories between 1989 to 2002

| LULC Classes | | 2002 | | | | | | | |
| --- | --- | --- | --- | --- | --- | --- | --- | --- | --- |
|  |  | Aquaculture | Creeks | Double  Cropland | Single cropland | Mangrove | Settlement | Vegetation | Waterbody |
| 1989 | Aquaculture | 0.552 | 0.032 | 0.017 | 0.028 | 0.011 | 0.028 | 0.023 | 0.098 |
|  | Creeks | 0.007 | 0.284 | 0.009 | 0.008 | 0.034 | 0.022 | 0.014 | 0.062 |
|  | Double cropland | 0.053 | 0.004 | 0.136 | 0.067 | 0.000 | 0.085 | 0.088 | 0.057 |
|  | Single cropland | 0.355 | 0.194 | 0.699 | 0.845 | 0.027 | 0.543 | 0.441 | 0.545 |
|  | Mangrove | 0.008 | 0.457 | 0.004 | 0.008 | 0.924 | 0.005 | 0.010 | 0.016 |
|  | Settlement | 0.004 | 0.004 | 0.021 | 0.004 | 0.000 | 0.064 | 0.062 | 0.049 |
|  | Vegetation | 0.014 | 0.013 | 0.104 | 0.034 | 0.002 | 0.234 | 0.348 | 0.122 |
|  | Waterbody | 0.007 | 0.011 | 0.010 | 0.005 | 0.001 | 0.019 | 0.013 | 0.051 |

**Supplementary Table 4** Transitional matrix of LULC categories between 2002 to 2011

| LULC Classes | | 2011 | | | | | | | |
| --- | --- | --- | --- | --- | --- | --- | --- | --- | --- |
|  |  | Aquaculture | Creeks | Double  Cropland | Single cropland | Mangrove | Settlement | Vegetation | Waterbody |
| 2002 | Aquaculture | 0.79 | 0.04 | 0.01 | 0.02 | 0.00 | 0.01 | 0.02 | 0.13 |
|  | Creeks | 0.01 | 0.37 | 0.01 | 0.01 | 0.03 | 0.01 | 0.01 | 0.03 |
|  | Double cropland | 0.02 | 0.01 | 0.15 | 0.12 | 0.00 | 0.16 | 0.17 | 0.08 |
|  | Single cropland | 0.12 | 0.10 | 0.61 | 0.75 | 0.00 | 0.22 | 0.34 | 0.49 |
|  | Mangrove | 0.03 | 0.43 | 0.01 | 0.02 | 0.96 | 0.01 | 0.01 | 0.10 |
|  | Settlement | 0.01 | 0.01 | 0.06 | 0.03 | 0.00 | 0.11 | 0.08 | 0.04 |
|  | Vegetation | 0.01 | 0.02 | 0.14 | 0.05 | 0.00 | 0.46 | 0.35 | 0.08 |
|  | Waterbody | 0.01 | 0.01 | 0.01 | 0.01 | 0.00 | 0.01 | 0.02 | 0.05 |

**Supplementary Table 5** Transitional matrix of LULC categories between 2011 to 2015

| LULC Classes | | 2015 | | | | | | | |
| --- | --- | --- | --- | --- | --- | --- | --- | --- | --- |
|  |  | Aquaculture | Creeks | Double  Cropland | Single cropland | Mangrove | Settlement | Vegetation | Waterbody |
| 2011 | Aquaculture | 0.815 | 0.086 | 0.013 | 0.018 | 0.004 | 0.024 | 0.007 | 0.073 |
|  | Creeks | 0.006 | 0.296 | 0.002 | 0.003 | 0.013 | 0.008 | 0.003 | 0.025 |
|  | Double cropland | 0.012 | 0.022 | 0.145 | 0.118 | 0.001 | 0.181 | 0.172 | 0.132 |
|  | Single cropland | 0.141 | 0.202 | 0.611 | 0.773 | 0.010 | 0.455 | 0.343 | 0.562 |
|  | Mangrove | 0.003 | 0.331 | 0.006 | 0.003 | 0.965 | 0.002 | 0.004 | 0.009 |
|  | Settlement | 0.001 | 0.006 | 0.062 | 0.020 | 0.001 | 0.107 | 0.175 | 0.040 |
|  | Vegetation | 0.014 | 0.042 | 0.157 | 0.060 | 0.002 | 0.216 | 0.293 | 0.143 |
|  | Waterbody | 0.008 | 0.016 | 0.005 | 0.004 | 0.004 | 0.006 | 0.004 | 0.014 |

**Supplementary Table 6** Transitional matrix of LULC categories between 2015 to 2019

| LULC Classes | | 2019 | | | | | | | |
| --- | --- | --- | --- | --- | --- | --- | --- | --- | --- |
|  |  | Aquaculture | Creeks | Double  Cropland | Single cropland | Mangrove | Settlement | Vegetation | Waterbody |
| 2015 | Aquaculture | 0.777 | 0.022 | 0.009 | 0.034 | 0.001 | 0.010 | 0.007 | 0.101 |
|  | Creeks | 0.005 | 0.311 | 0.004 | 0.011 | 0.013 | 0.004 | 0.004 | 0.076 |
|  | Double cropland | 0.015 | 0.014 | 0.276 | 0.083 | 0.001 | 0.150 | 0.165 | 0.096 |
|  | Single cropland | 0.170 | 0.107 | 0.552 | 0.747 | 0.007 | 0.420 | 0.398 | 0.361 |
|  | Mangrove | 0.017 | 0.502 | 0.015 | 0.008 | 0.977 | 0.008 | 0.010 | 0.260 |
|  | Settlement | 0.006 | 0.019 | 0.039 | 0.040 | 0.000 | 0.101 | 0.085 | 0.027 |
|  | Vegetation | 0.006 | 0.016 | 0.096 | 0.068 | 0.001 | 0.294 | 0.319 | 0.061 |
|  | Waterbody | 0.003 | 0.008 | 0.009 | 0.011 | 0.000 | 0.012 | 0.011 | 0.018 |

**Supplementary Table 7** Transitional matrix of LULC categories between 2019 to 2022

| LULC Classes | | 2022 | | | | | | | |
| --- | --- | --- | --- | --- | --- | --- | --- | --- | --- |
|  |  | Aquaculture | Creeks | Double  Cropland | Single cropland | Mangrove | Settlement | Vegetation | Waterbody |
| 2019 | Aquaculture | 0.823 | 0.017 | 0.008 | 0.028 | 0.001 | 0.024 | 0.014 | 0.107 |
|  | Creeks | 0.002 | 0.250 | 0.003 | 0.003 | 0.013 | 0.006 | 0.004 | 0.068 |
|  | Double cropland | 0.014 | 0.018 | 0.322 | 0.108 | 0.003 | 0.090 | 0.097 | 0.075 |
|  | Single cropland | 0.130 | 0.114 | 0.471 | 0.741 | 0.007 | 0.551 | 0.452 | 0.568 |
|  | Mangrove | 0.020 | 0.535 | 0.004 | 0.003 | 0.970 | 0.003 | 0.003 | 0.081 |
|  | Settlement | 0.004 | 0.006 | 0.051 | 0.037 | 0.001 | 0.102 | 0.112 | 0.013 |
|  | Vegetation | 0.004 | 0.013 | 0.136 | 0.074 | 0.002 | 0.218 | 0.316 | 0.037 |
|  | Waterbody | 0.003 | 0.047 | 0.006 | 0.005 | 0.002 | 0.006 | 0.004 | 0.050 |

**Supplementary Table 8** Transitional matrix of LULC categories between 2019 to 2049

| LULC Classes | | 2049 | | | | | | | |
| --- | --- | --- | --- | --- | --- | --- | --- | --- | --- |
|  |  | Aquaculture | Creeks | Double  Cropland | Single cropland | Mangrove | Settlement | Vegetation | Waterbody |
| 2019 | Aquaculture | 0.744 | 0.002 | 0.001 | 0.005 | 0.000 | 0.003 | 0.001 | 0.001 |
|  | Creeks | 0.001 | 0.586 | 0.002 | 0.001 | 0.008 | 0.002 | 0.001 | 0.005 |
|  | Double cropland | 0.034 | 0.016 | 0.807 | 0.022 | 0.000 | 0.098 | 0.062 | 0.099 |
|  | Single cropland | 0.137 | 0.060 | 0.111 | 0.936 | 0.001 | 0.228 | 0.120 | 0.074 |
|  | Mangrove | 0.003 | 0.318 | 0.001 | 0.001 | 0.990 | 0.003 | 0.001 | 0.034 |
|  | Settlement | 0.030 | 0.004 | 0.023 | 0.014 | 0.000 | 0.449 | 0.058 | 0.019 |
|  | Vegetation | 0.048 | 0.012 | 0.050 | 0.019 | 0.000 | 0.213 | 0.755 | 0.044 |
|  | Waterbody | 0.002 | 0.002 | 0.005 | 0.001 | 0.001 | 0.004 | 0.003 | 0.725 |

**Supplementary Table 9** Transitional matrix of salinity classes between 2019 to 2049

| Salinity Classes | | 2049 | | |
| --- | --- | --- | --- | --- |
| 2019 |  | Non-saline | Saline | Slightly saline |
|  | Non-saline | 0.617 | 0.046 | 0.158 |
|  | Saline | 0.099 | 0.863 | 0.341 |
|  | Slightly saline | 0.284 | 0.091 | 0.501 |
